# Supplementary material for: PINES: phenotype-informed tissue weighting improves prediction of pathogenic noncoding variants
Source: Genome Biol. 2018 Oct 25;19:173. doi: 10.1186/s13059-018-1546-6 (PMC6203199; doi:10.1186/s13059-018-1546-6)
Supplement: Supplementary file 1 — Supplementary information. (PDF 8936 kb) [file 13059_2018_1546_MOESM1_ESM.pdf]

PINES: phenotype-informed tissue weighting improves prediction of pathogenic noncoding variants

Corneliu A. Bodea[1,2,4], Adele A. Mitchell[1], Alex Bloemendal[4], Aaron G. Day-Williams[1‡*], Heiko Runz[1‡*], Shamil R. Sunyaev[2,3,4‡*]

1 Department of Genetics and Pharmacogenomics, MRL, Boston, Massachusetts, USA

2 Division of Genetics, Department of Medicine, Brigham and Women's Hospital and Harvard Medical School, Boston, Massachusetts, USA

3 Department of Biomedical Informatics, Harvard Medical School, Boston, Massachusetts, USA

4 The Broad Institute of MIT and Harvard, Cambridge, Massachusetts, USA

‡These authors jointly supervised this work.

* ssunyaev@rics.bwh.harvard.edu, heiko.runz@gmail.com, or aaron.day-williams@merck.com

Supplemental Material

Variant	Phenotype	Target gene	Role
rs12350739	Pigmentation	BNC2	rs12350739 falls within a highly conserved region that functions as an enhancer element regulating BNC2 transcription in human melanocytes, and the activity of this enhancer depends on the allelic status of rs12350739.
rs356168	Parkinson's disease	SNCA	rs356168 affects enhancer activity regulating the expression of SNCA.
rs6801957	QRS prolongation	SCN5A	rs6801957 affects the activity of an intronic enhancer regulation the expression of the nearby gene SCN5A.
rs12821256	Pigmentation	KITLG	rs12821256 alters a LEF1 binding site, which reduces LEF1 responsiveness and enhancer activity on KITLG in cultured human keratinocytes.
rs227727	Nonsyndromic cleft lip with or without cleft palate	NOG	rs227727 alters enhancer activity for NOG, a gene involved in the development of nerve tissue, muscles, and bones.
rs2473307	Schizophrenia	CDC42	rs2473307 alters enhancer activity for CDC42 and leads to reduced expression of the gene.

Table S1. Summary of experimentally validated noncoding variants.

PINES lead variants: inflammatory bowel disease					
**rs ID**	**Risk allele**	**Position**	**Target gene**	**Regulatory element**	**Proposed biology**
**rs35493230**	G	Upstream of SPRED2	SPRED2	Promoter	rs35493230 lies in the promoter region of SPRED2, a gene also implicated in GWAS of rheumatoid arthritis and a member of a family of proteins that regulate growth factor-induced activation of the MAP kinase cascade. rs35493230 is an eQTL for SPRED2 and overlaps with a POLR2A binding site upstream of SPRED2.
**rs2187892**	A	Intergenic	TNFSF4	Enhancer	rs2187892 shows Hi-C contact to the promoter of TNFSF4 about 300kb away, which encodes a cytokine of the tumor necrosis factor ligand family. The encoded protein functions in T cell antigen-presenting cell interactions, and mediates adhesion of activated T-cells to endothelial cells. Functional analysis showed that anti-TNFSF4 monoclonal antibody inhibited T-cell proliferation and the development of colitis in mice.
**rs4672507**	T	Intergenic	COMMD1	Enhancer	rs4672507 shows Hi-C contact to loci in COMMD1. This gene is a negative regulator of NF-kB, and recent genetic studies in both mice and human patients indicate that COMMD1 plays an important role in controlling intestinal inflammation and constraining progression to colitis-associated cancer.
**rs4845604**	G	Intron of RORC	RORC	Enhancer	rs4845604 is an eQTL for RORC in whole blood. RORC encodes a DNA-binding transcription factor and contains autosomal recessive variants for immunodeficiency. The protein encoded by RORC, and the Tregs that express it, contribute substantially to regulating colonic TH1/TH17 inflammation. ENCODE ChIP-Seq experiments report binding of SIN3A, REST, and SP2 to the rs4845604 locus.
**rs2019262**	G	Intron of IL23R	IL23R	Enhancer	rs2019262 overlaps with several transcription factor binding sites in an intron of IL23R, a known IBD gene involved in IL23 signaling.
**rs10489630**	A	Intron of IL23R	IL23R	Enhancer	rs10489630 falls within an intron of IL23R, a known IBD gene involved in IL23 signaling.
**rs12622128**	A	Upstream of IL1R1, downstream of IL1R2	Unclear: IL1R1, IL1R2	Enhancer	rs12622128 is an eQTL for IL1R2, a gene involved in IL1 signaling, and overlaps a GATA2 binding site.
**rs55776317**	C	Intron of PUS10	Unclear: PUS10, REL	Enhancer	rs55776317 and rs7585642 fall within an intron of PUS10, a gene involved in pseudouridination of RNA. This locus also shows Hi-C contact to the transcription start site of REL, a gene involved in apoptosis, inflammation, the immune response, and oncogenic processes. Single nucleotide polymorphisms in this gene are associated with susceptibility to ulcerative colitis and rheumatoid arthritis.
**rs7585642**	C	Intron of PUS10	Unclear: PUS10, REL	Enhancer	

Table S2. Novel noncoding pathogenic variants identified by PINES functional prioritization at inflammatory bowel disease loci. For each lead SNP, all variants with $LD \geq 0.4$ were selected, and loci were discarded if this list overlapped any coding regions or 3' or 5' UTRs. The remaining loci are likely regulatory in nature, so all variants in LD to the lead SNP were scored via weighted PINES. The lead SNPs from loci where a peak was identified are presented.

PINES lead variants: Parkinson's disease					
**rs ID**	**Risk allele**	**Position**	**Target gene**	**Regulatory element**	**Proposed biology**
**rs10878226**	C	Upstream of LRRK2	LRRK2	Promoter	rs10878226 overlaps with a POLR2A binding site in the promoter region of LRRK2, a known Parkinson's disease gene harboring multiple autosomal dominant disease variants. This variant is also an eQTL of LRRK2.
**rs3756063**	C	Intron of SNCA	SNCA	Promoter	rs3756063 is an eQTL of SNCA, a known Parkinson's disease gene. This variant was reported to affect promoter hypomethylation of SNCA, and overlaps several transcription factor binding sites.
**rs2301134**	G	Intron of SNCA	SNCA	Promoter	rs2301134 is an eQTL of SNCA, a known Parkinson's disease gene. ENCODE ChIP-Seq experiments report binding of CTCF, POLR2A, RAD21, and EZH2 to the rs2301134 locus.
**rs36121867**	T	Intron of FAM47E	SCARB2	Enhancer	rs36121867 is an eQTL for SCARB2, a gene harboring autosomal recessive variants for progressive myoclonic epilepsy. SCARB2-deficient mice were also characterized by peripheral demyelinating neuropathy. Hi-C data shows interaction between the rs36121867 locus and the SCARB2 promoter, suggesting enhancer activity for this variant.
**rs1954874**	C	Upstream of ACMSD	ACMSD	Promoter	rs1954874 lies in the promoter of ACMSD, an enzyme that prevents the accumulation of the neuronal excitotoxin quinolinate as part of the synthesis of NAD from tryptophan. Quinolinate has been implicated in the pathogenesis of several neurodegenerative disorders. ACMSD was also found to be mutated in a family with cortical myoclonus, epilepsy, and Parkinsonism.
**rs9275373**	G	Intergenic	Unclear	Enhancer	rs9275373 is an eQTL for several HLA genes that fall in proximity to this variant (HLA–DQA1, HLA–DQA2, HLA–DQB1, HLA–DQB2, HLA–DRB1). The involvement of the immune system in Parkinson's disease is currently under investigation, and the brains of individuals with Parkinson's disease show upregulation of DR antigens and the presence of DR-positive reactive microglia.
**rs117896735**	A	Intron of INPP5F	BAG3	Enhancer	rs117896735 shows Hi-C contact to the transcription start site of BAG3, a gene implicated in age related neurodegenerative diseases and giant axonal neuropathy.

Table S3. Novel noncoding pathogenic variants identified by PINES functional prioritization at Parkinson's disease loci. For each lead SNP, all variants with $LD \geq 0.4$ were selected, and loci were discarded if this list overlapped any coding regions or 3' or 5' UTRs. The remaining loci are likely regulatory in nature, so all variants in LD to the lead SNP were scored via weighted PINES. The lead SNPs from loci where a peak was identified are presented.

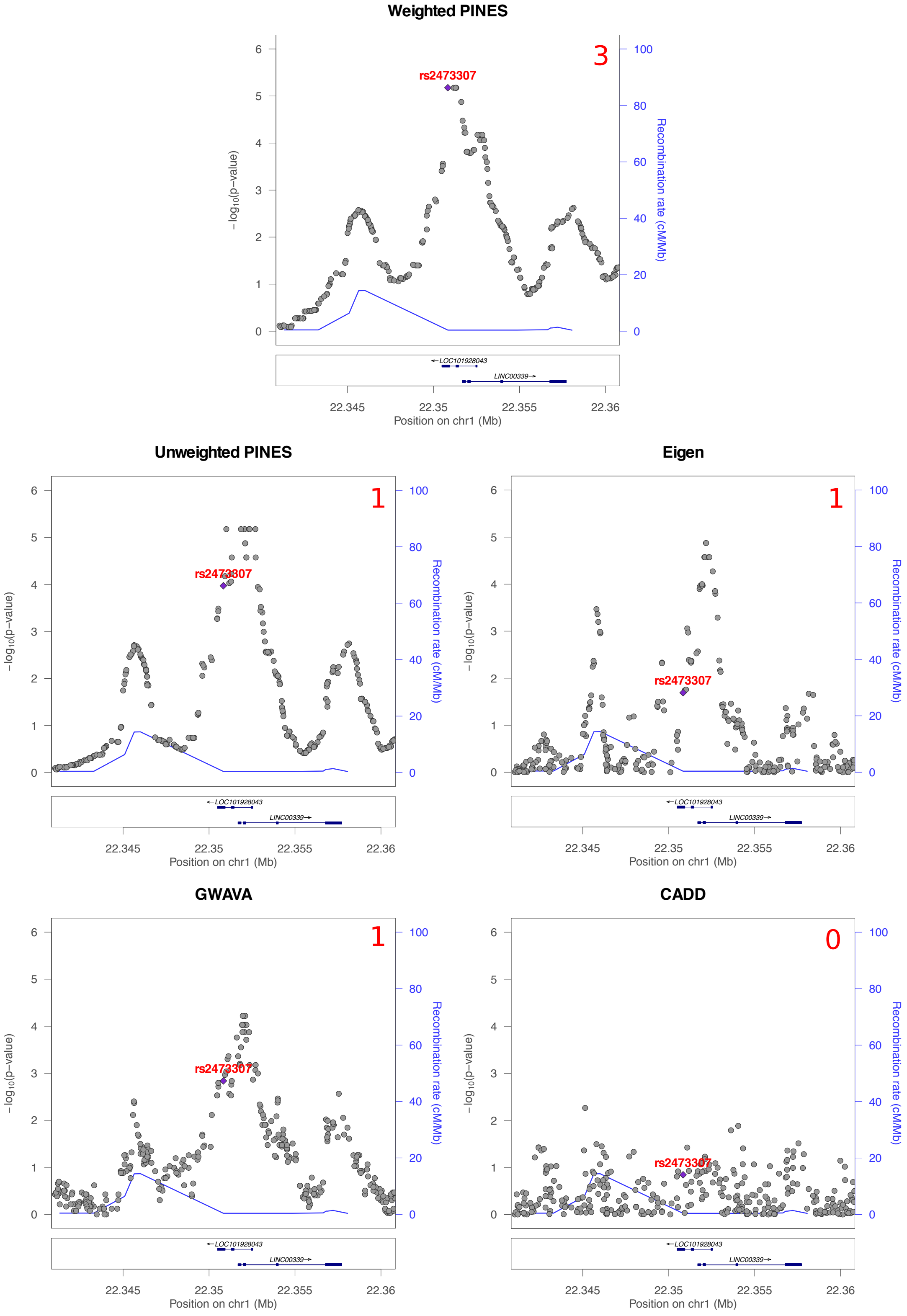

Figure S1. Variant prioritization at the 20kb region surrounding the schizophrenia risk variant rs2473307. PINES achieves the best prioritization. The values in the upper corners of the plot assign up to 3 points for each method for the presence of a peak around the known functional variant, the ranking of the functional variant in the top 5 scores in the peak, and the method achieving the strongest significance level relative to the other three.

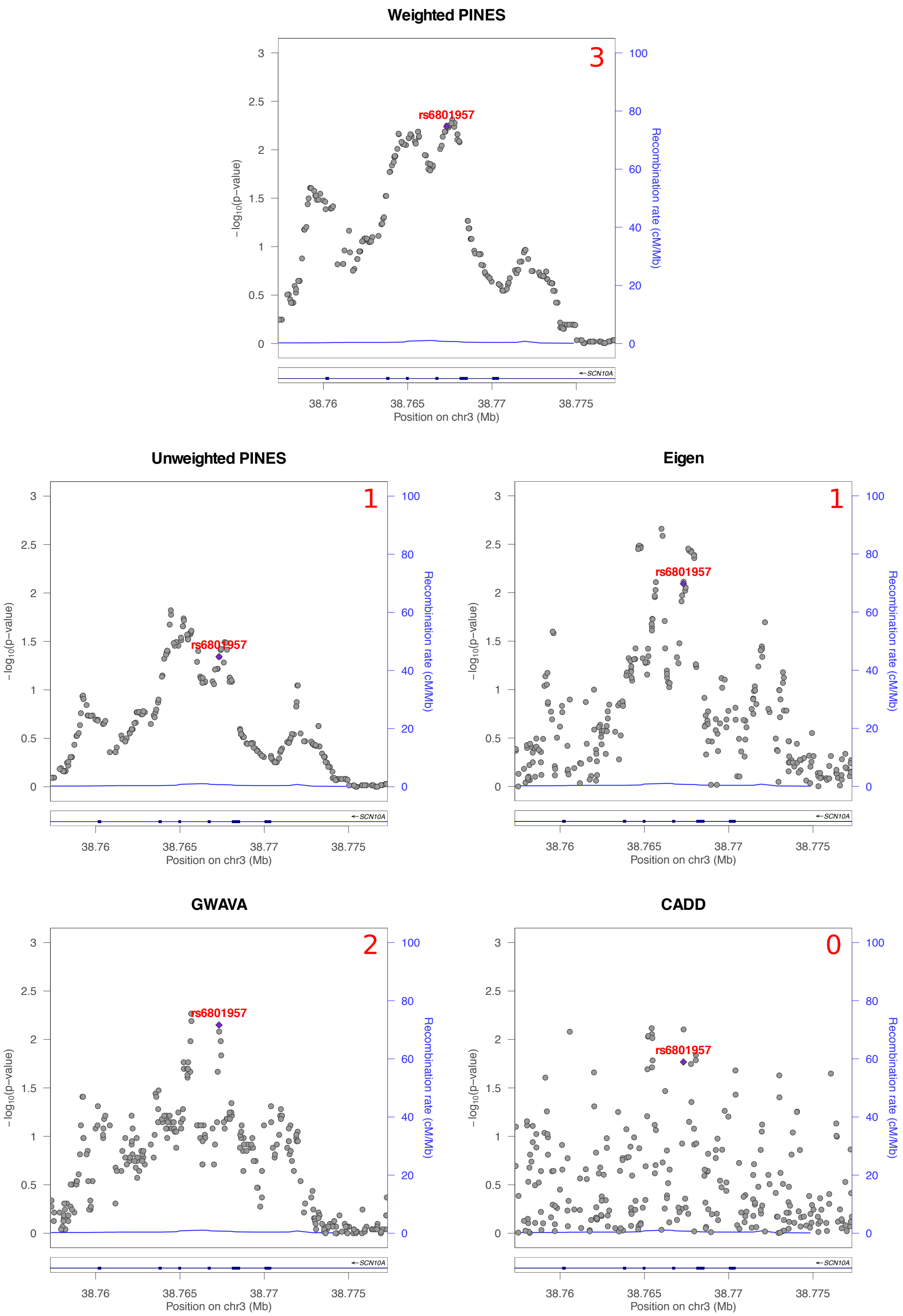

Figure S2. Variant prioritization at the 20kb region surrounding the QRS duration variant rs6801957. PINES achieves the best prioritization. The values in the upper corners of the plot assign up to 3 points for each method for the presence of a peak around the known functional variant, the ranking of the functional variant in the top 5 scores in the peak, and the method achieving the strongest significance level relative to the other three.

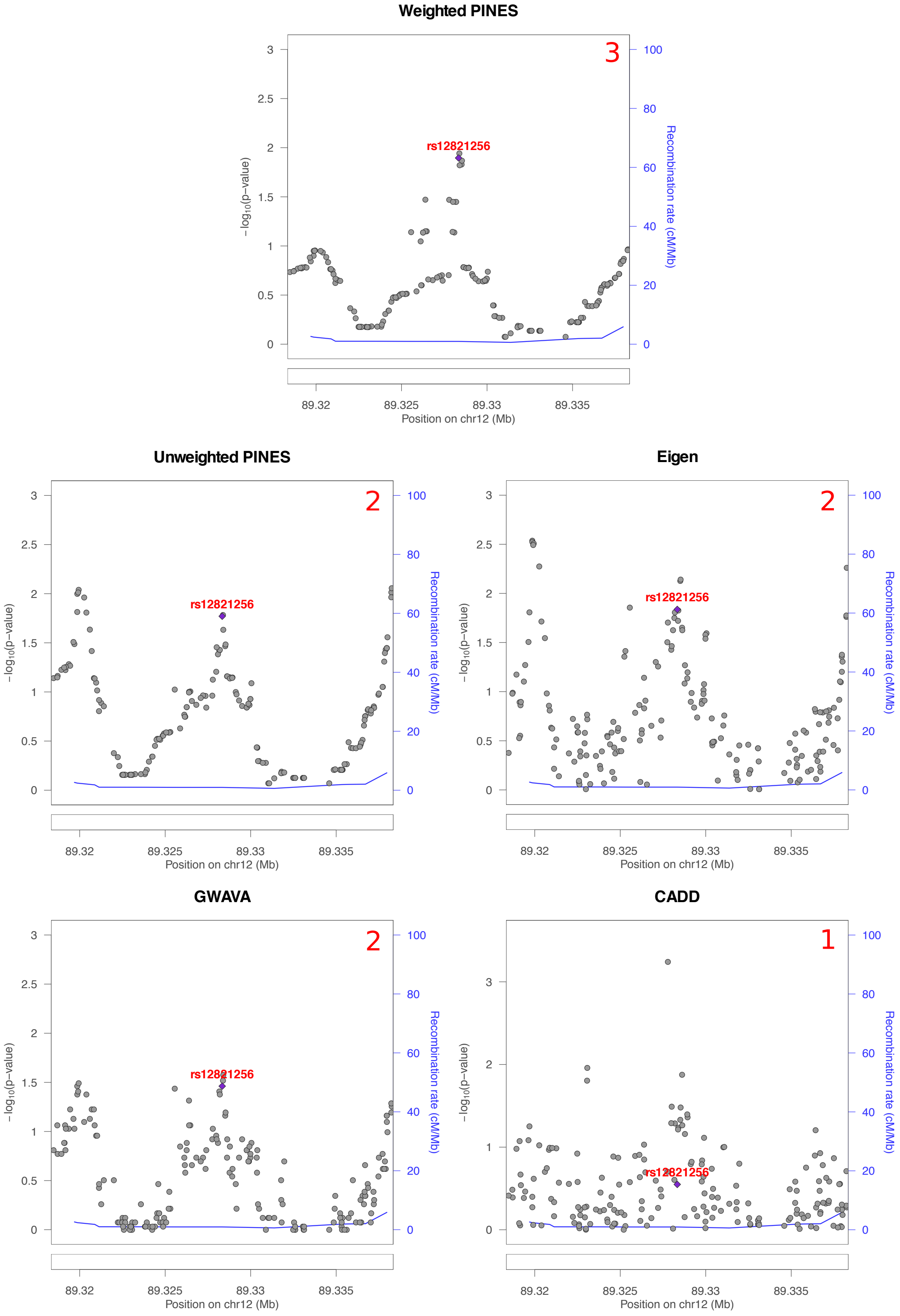

Figure S3. Variant prioritization at the 20kb region surrounding the pigmentation variant rs12821256. This SNP alters a LEF1 binding site, which reduces LEF1 responsiveness and enhancer activity on KITLG in cultured human keratinocytes. PINES achieves the best prioritization and brings focus to the relevant peak. The values in the upper corners of the plot assign up to 3 points for each method for the presence of a peak around the known functional variant, the ranking of the functional variant in the top 5 scores in the peak, and the method achieving the strongest significance level relative to the other three.

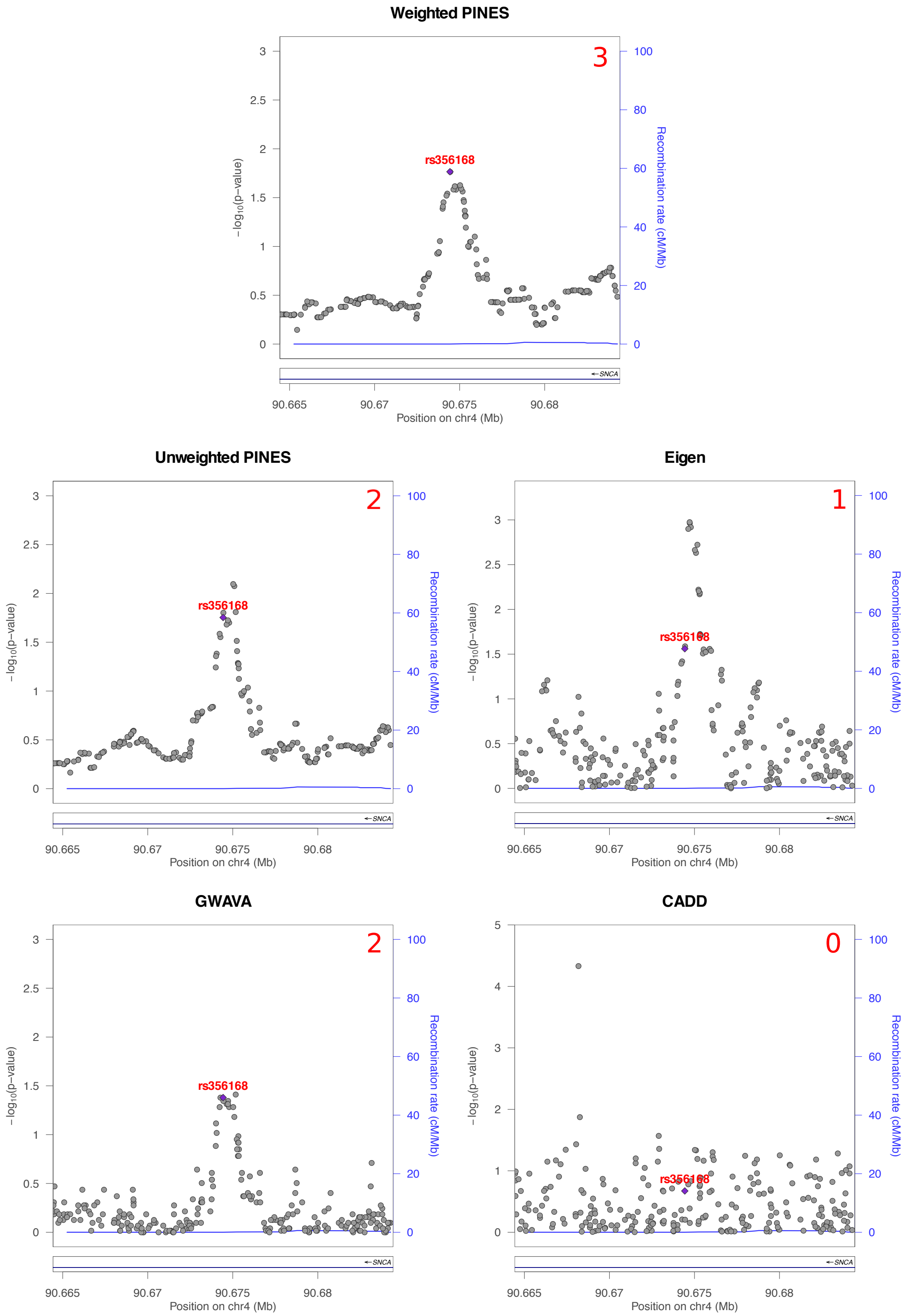

Figure S4. Variant prioritization at the 20kb region surrounding the Parkinson's disease risk variant rs356168. This SNP affects enhancer activity regulating the expression of SNCA. PINES achieves the best prioritization. The values in the upper corners of the plot assign up to 3 points for each method for the presence of a peak around the known functional variant, the ranking of the functional variant in the top 5 scores in the peak, and the method achieving the strongest significance level relative to the other three.

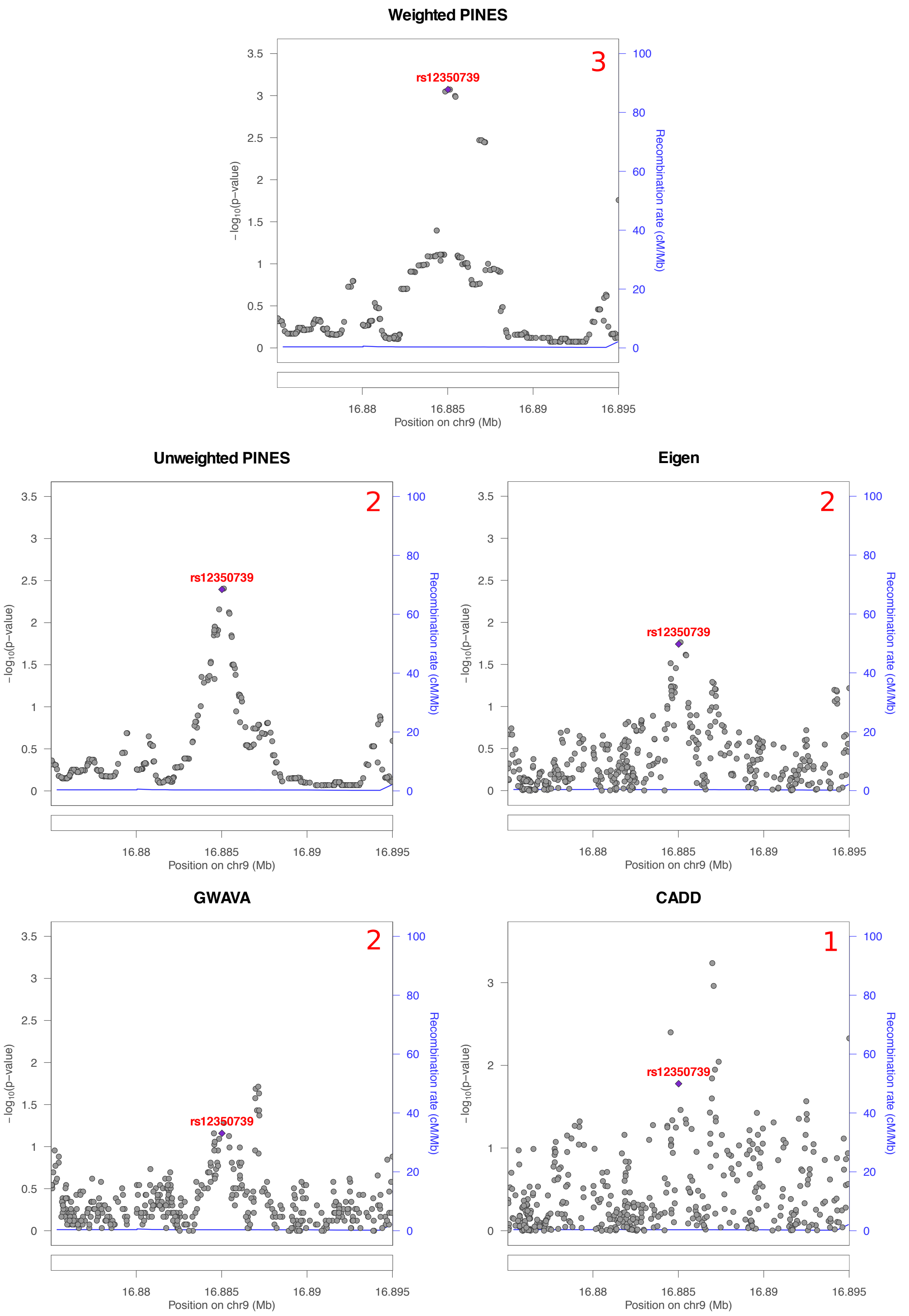

Figure S5. Variant prioritization at the 20kb region surrounding the intergenic pigmentation variant rs12350739. This SNP disrupts enhancer activity regulating the expression of BNC2 in human melanocytes. Enhancer activity is modulated by the allelic status of rs12350739 through changes in chromatin accessibility. PINES achieves the best prioritization. The values in the upper corners of the plot assign up to 3 points for each method for the presence of a peak around the known functional variant, the ranking of the functional variant in the top 5 scores in the peak, and the method achieving the strongest significance level relative to the other three.

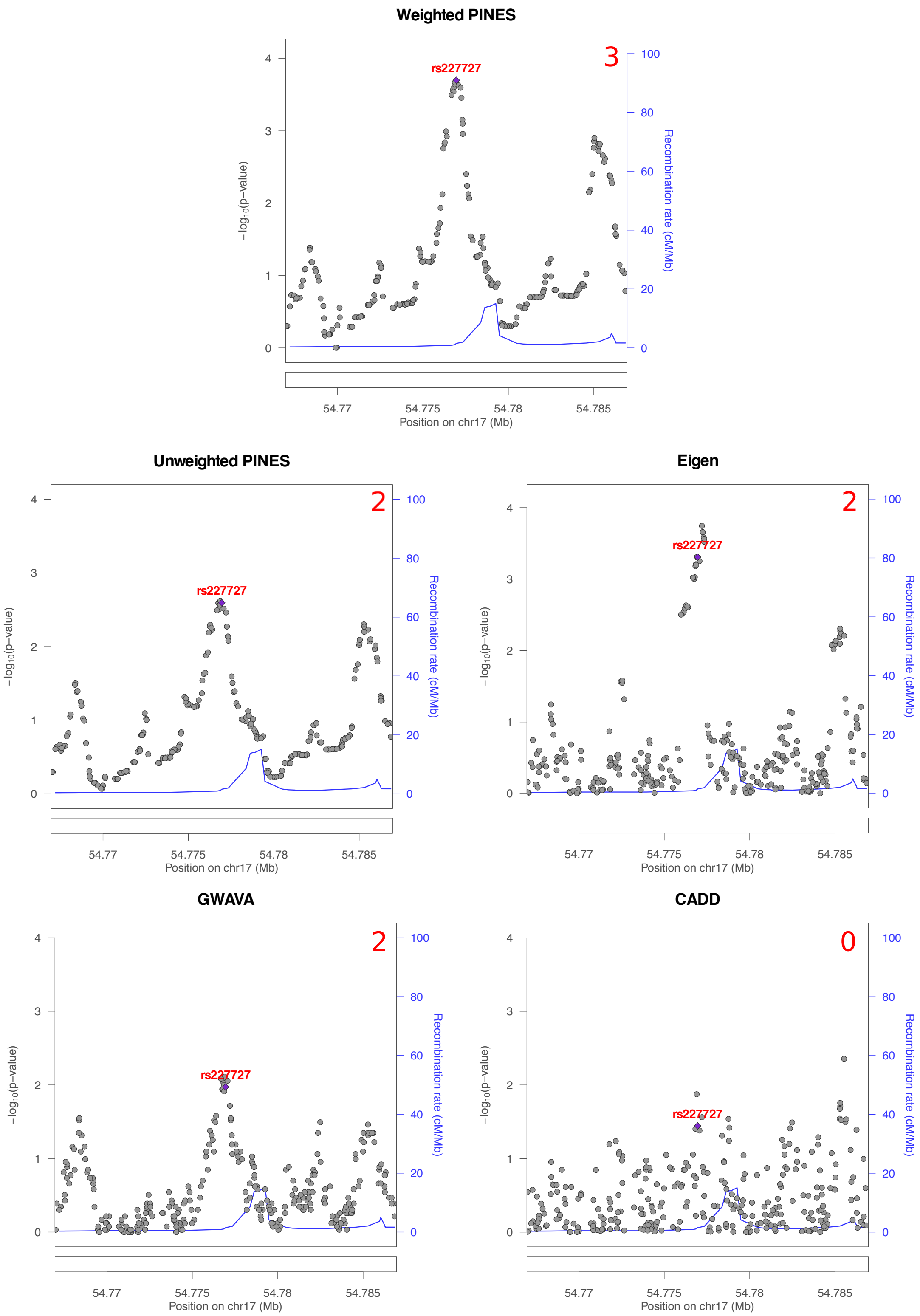

Figure S6. Variant prioritization at the 20kb region surrounding the nonsyndromic cleft lip with or without cleft palate risk variant rs227727. PINES achieves the best prioritization. The values in the upper corners of the plot assign up to 3 points for each method for the presence of a peak around the known functional variant, the ranking of the functional variant in the top 5 scores in the peak, and the method achieving the strongest significance level relative to the other three.

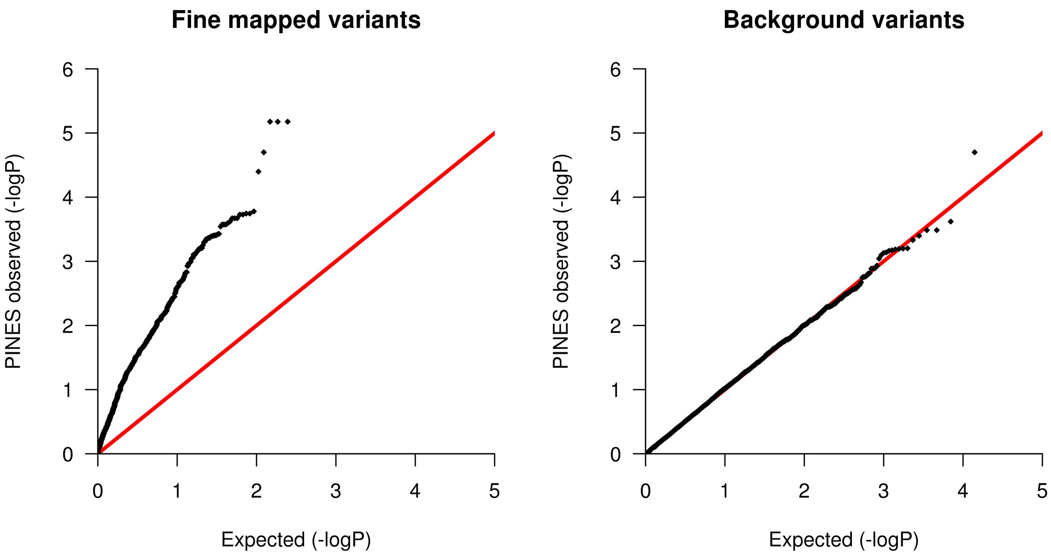

Figure S7. Comparison of PINES signal on a collection of immune-implicated variants that have been fine-mapped via PICS (left panel) versus a set of background variants (right panel). The null distribution measured on background variants is well calibrated, and ample signal is detected by PINES on the fine-mapped collection.

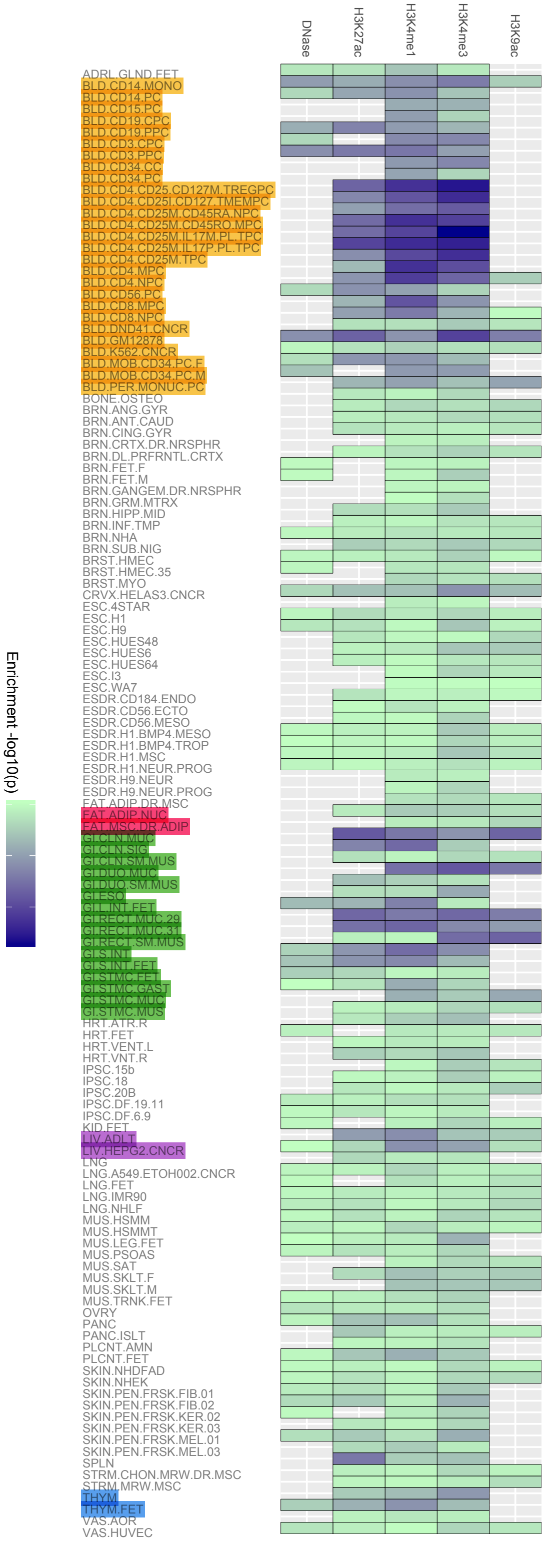

Figure S8. Weight matrix computed by PINES based on enrichment of epigenetic annotations across lead SNPs at inflammatory bowel disease GWAS loci. Annotations corresponding to cell types related to immunity and GI are upweighted, which will be reflected in all PINES scores computed based on this weight matrix. Cell types are color-coded as follows: immune - yellow, GI - green, thymus - blue, liver - purple, adipocytes - pink.

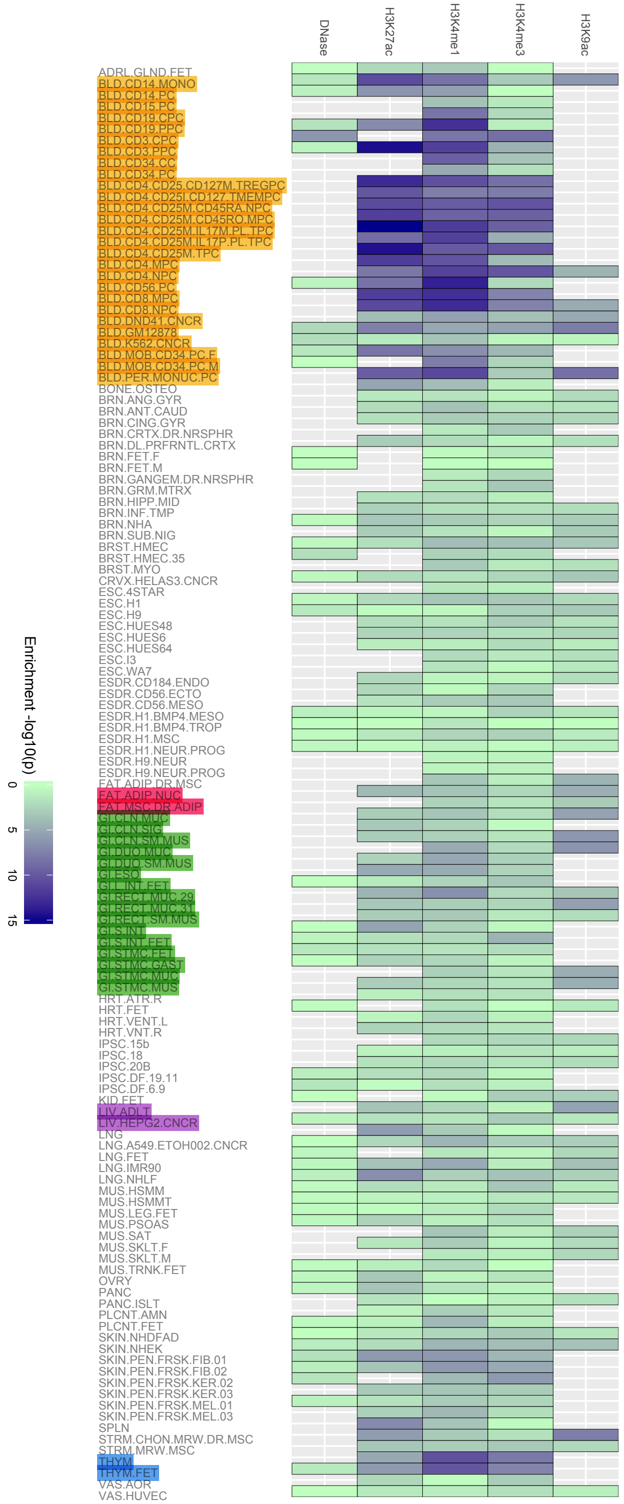

Figure S9. Weight matrix computed by PINES based on enrichment of epigenetic annotations across lead SNPs at multiple sclerosis GWAS loci. Annotations corresponding to cell types related to immunity and thymus are upweighted, which will be reflected in all PINES scores computed based on this weight matrix. Cell types are color-coded as follows: immune - yellow, GI - green, thymus - blue, liver - purple, adipocytes - pink.

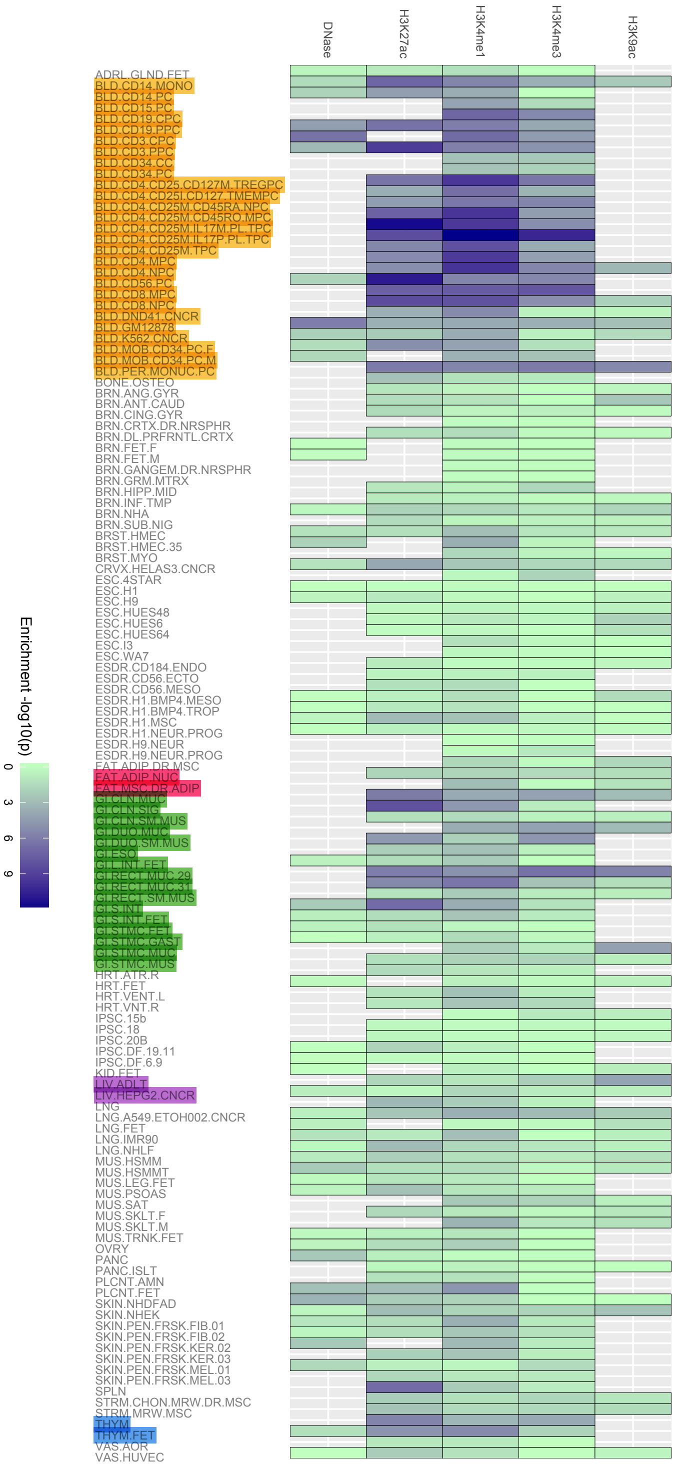

Figure S10. Weight matrix computed by PINES based on enrichment of epigenetic annotations across lead SNPs at celiac disease GWAS loci. Annotations corresponding to cell types related to immunity and GI are upweighted, which will be reflected in all PINES scores computed based on this weight matrix. Cell types are color-coded as follows: immune - yellow, GI - green, thymus - blue, liver - purple, adipocytes - pink.

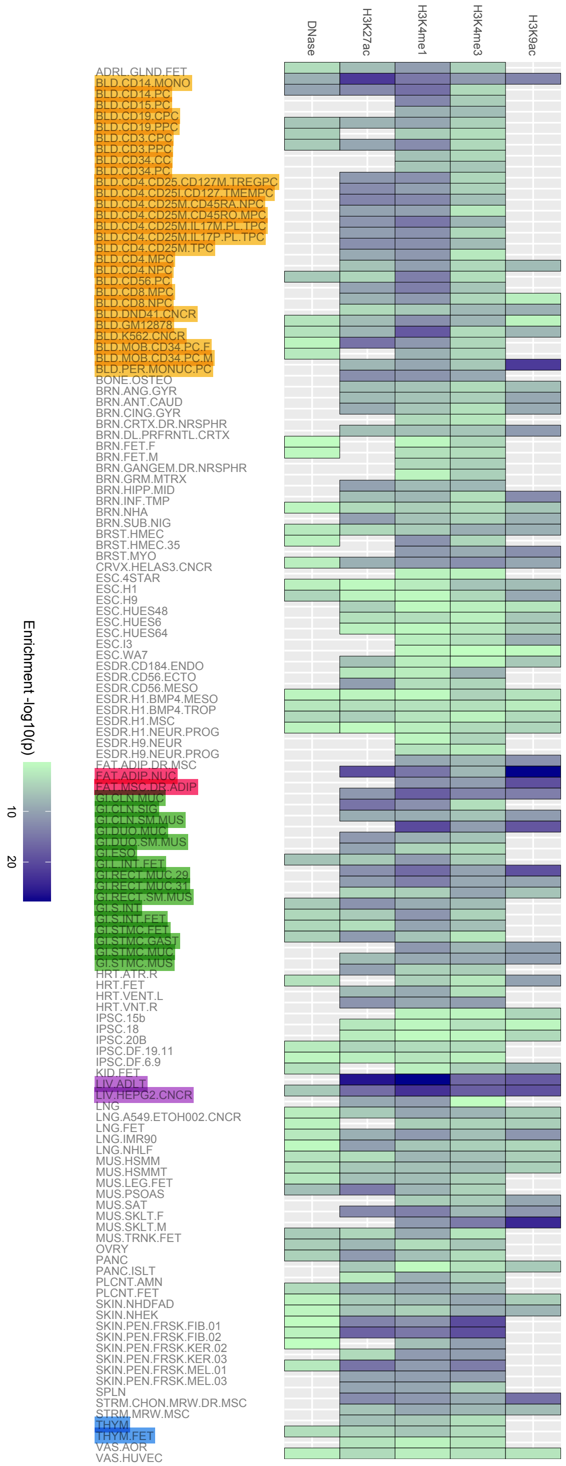

Figure S11. Weight matrix computed by PINES based on enrichment of epigenetic annotations across lead SNPs at blood lipids GWAS loci. Annotations corresponding to adipocytes and liver are upweighted, which will be reflected in all PINES scores computed based on this weight matrix. Cell types are color-coded as follows: immune - yellow, GI - green, thymus - blue, liver - purple, adipocytes - pink.

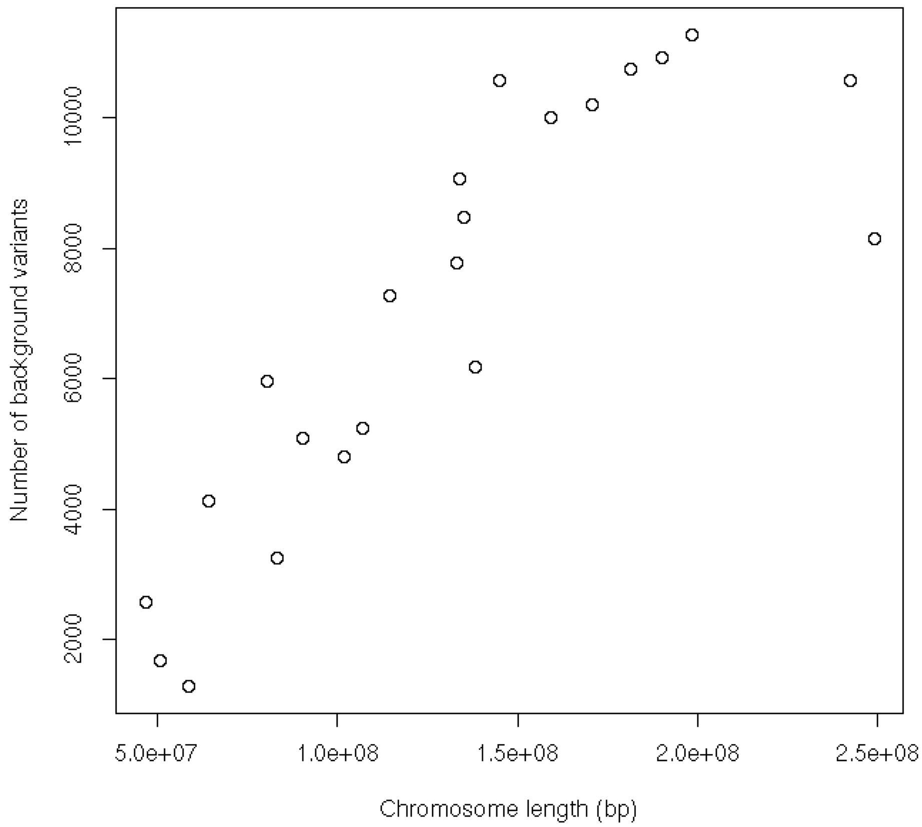

Figure S12. Relationship between the number of background variants on each chromosome and chromosome length.

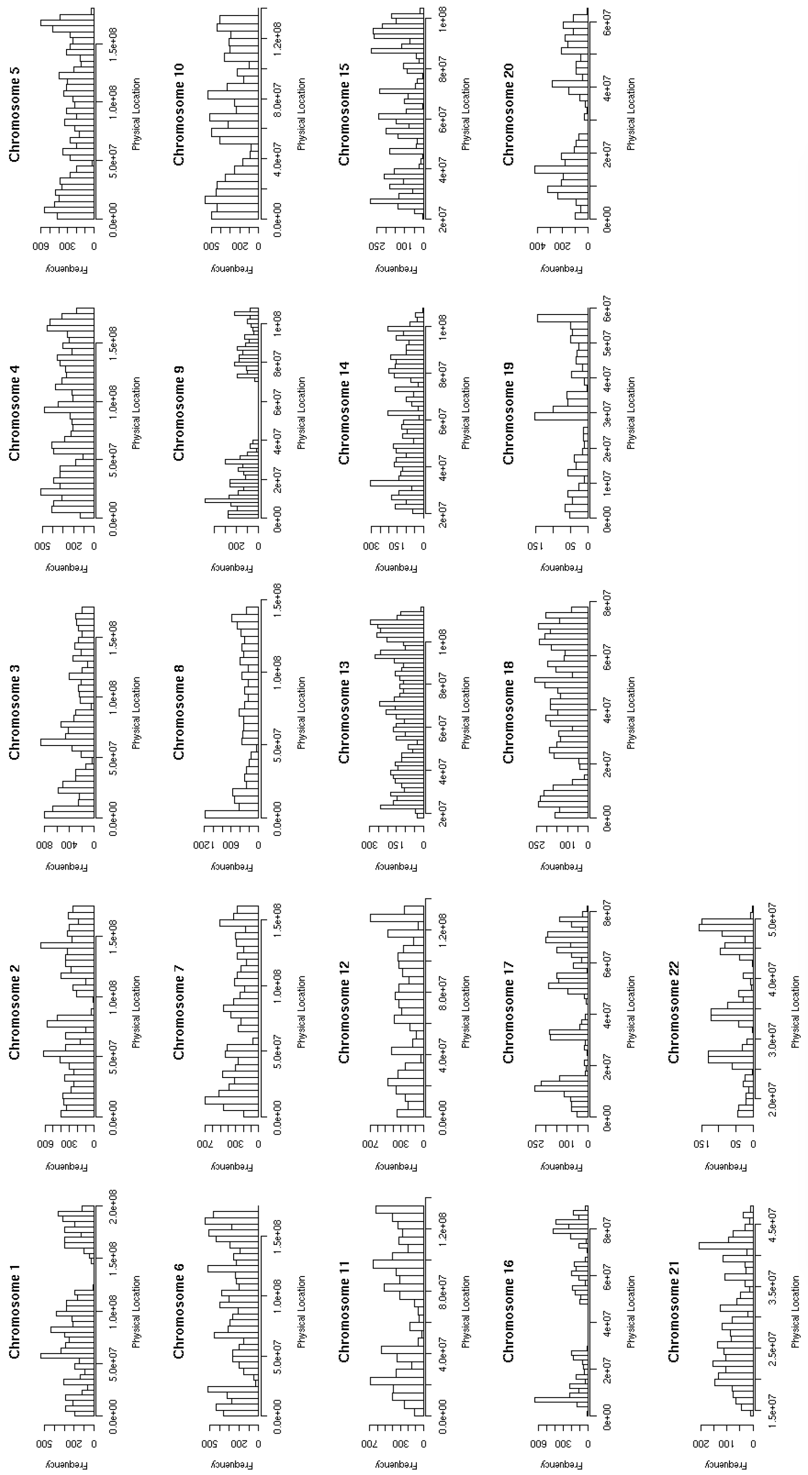

Figure S13. Distribution of background variants across autosomes.

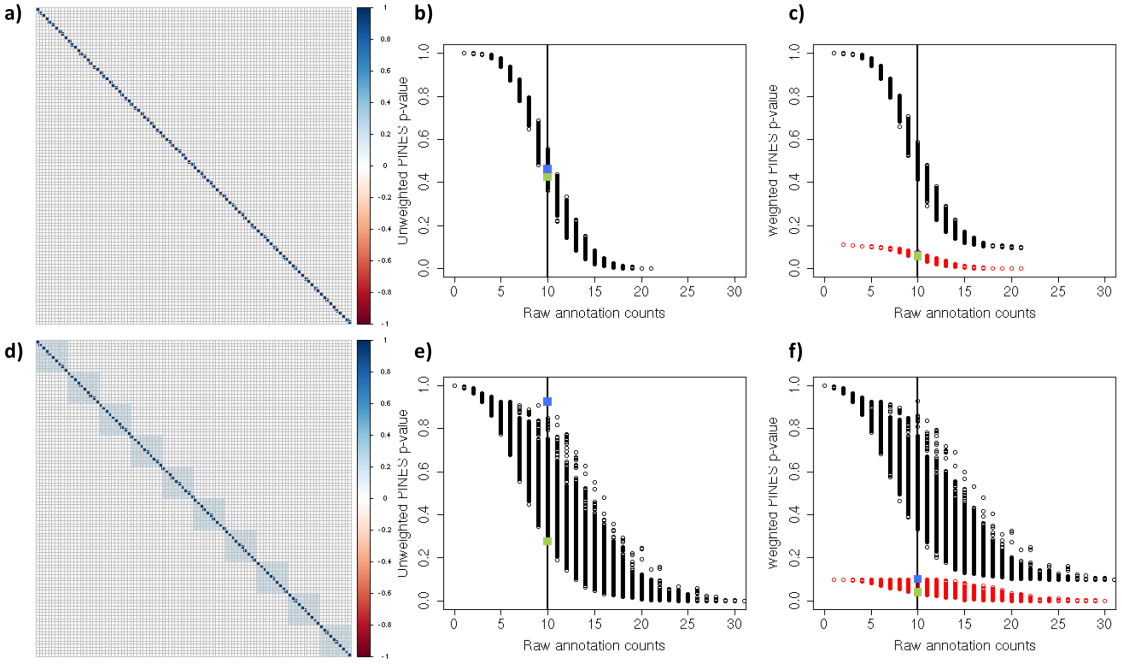

Figure S14. Simulation results depicting the behavior of PINES scores versus raw annotation counts. Variants are simulated under two scenarios: annotations are uncorrelated (panels a-c), or annotations exhibit correlation structure (panels d-f). Panels b and e highlight the behavior of unweighted PINES scores, and panels c and f highlight the behavior of weighted PINES scores where variants marked in red overlap the up-weighted annotation. For panels d-f we also highlight two variants: the variant that overlaps the first annotation from each of the 10 blocks of correlated annotations (green), and the variant that overlaps all ten annotations from the first block of correlated annotations (blue). Thus, both the green and the blue variant overlap ten annotations, but the selected annotations reflect different correlation patterns (uncorrelated annotations for green, correlated annotations for blue). For panels a-c the same selection of variants and color-coding is employed, but now all annotations are uncorrelated.

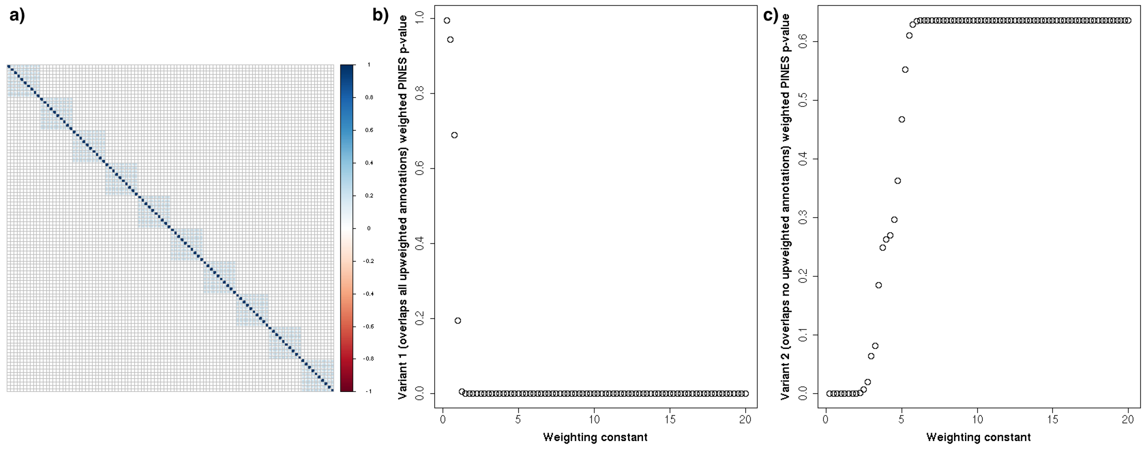

Figure S15. Dynamics of weighted PINES scores in simulated data as the weighting constant increases.

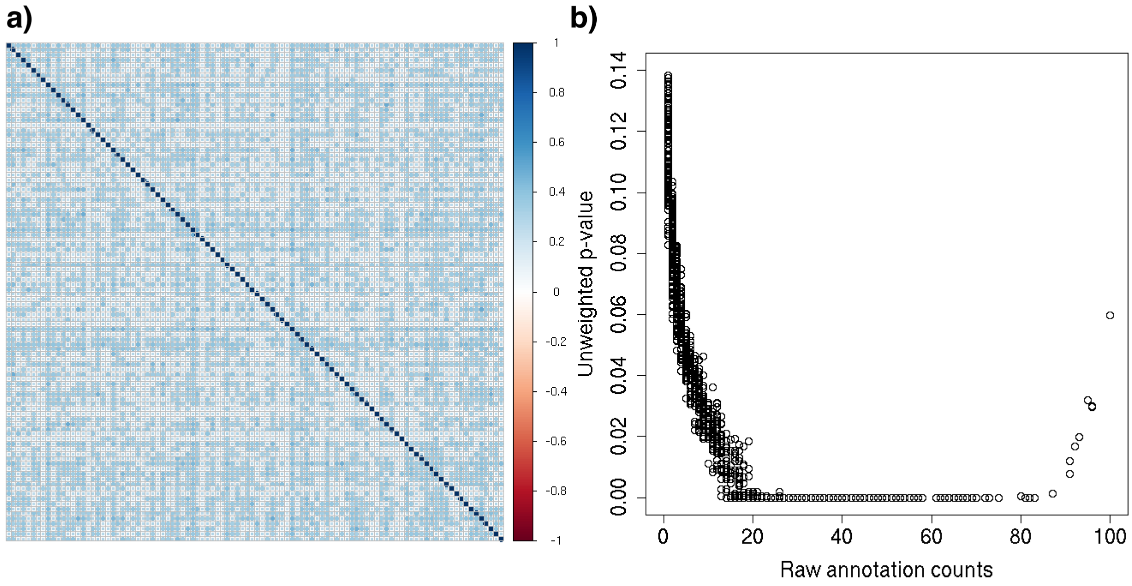

Figure S16. Dynamics of p-values resulting from only on a Cholesky transformation of the epigenetic annotation space.

Method	Number of loci for which method achieved the most significant average p-value
Unweighted PINES	15
Eigen-PC	10
GWAVA (region matched)	10
LINSIGHT	10
DANN	7
GWAVA (TSS matched)	7
GWAVA (unmatched)	7
FATHMM-MKL	3
CADD	2

Table S4. Counts represent the number of loci for which each method achieved the most significant average p-value.
