# Supplementary material for: PINES: phenotype-informed tissue weighting improves prediction of pathogenic noncoding variants
Source: Genome Biol. 2018 Oct 25;19:173. doi: 10.1186/s13059-018-1546-6 (PMC6203199; doi:10.1186/s13059-018-1546-6)
Supplement: Supplementary file 2 — Review history. (DOCX 73 kb) [file 13059_2018_1546_MOESM2_ESM.docx]

Review History

**First round of review**

**Reviewer 1**

**Are the methods appropriate to the aims of the study, are they well described, and are necessary controls included?**
Yes.

**Are the conclusions adequately supported by the data shown?**

Mostly

**Are sufficient details provided to allow replication and comparison with related analyses that may have been performed?**

Not sure, it is not stated in the paper that all the data are made available.

**Is the method likely to be of broad utility? Is any software component easy to install and use?**

Potentially yes, if the method indeed perform as the authors claimed.

**Is the paper of broad interest to others in the field, or of outstanding interest to a broad audience of biologists?**

Yes: important for human genetics.

Comments to authors:

Bodea et al. describe the computational framework PINES (Phenotype-Informed Noncoding Element Scoring) which evaluates the functional impact of noncoding variants in an unsupervised setting by integrating epigenetic annotations from diverse sources in a phenotype dependent manner. The authors showed PINES enjoys superior performance when comparing to competing methods such as CADD. PINES can also help interpret the results of finding mapping studies and predict novel causal alleles of complex diseases. Here are my comments. 

1. Overall, I feel PINES has interesting ideas that differ from most of existing methods. It focuses on noncoding variants, unsupervised, and uses cell type specific features. On the other hand, all of these have been implemented in one of more existing methods, albeit not in the same one. Since only a limited number of phenotypes is tested, it is unclear how significant is this new method. 

2. The authors claim that PINES is "unsupervised". But they state that "A unique feature of PINES is that analyses may be customized towards genomic annotations of the highest relevance to phenotypes of interest." Such annotations include GWAS-implicated region to serve as positive control. I feel that these two statements contradict each other. In fact, the "weighted" version of PINES is clearly supervised. 

3. There are a couple of recent studies, namely GenoCanyon, a whole-genome functional annotation approach based on unsupervised statistical learning (Lu et al. Scientific Reports 2015); and DIVAN, a disease-specific, non-coding variant annotation tool (Chen et al. Genome Biology 2016). I feel that there are some similarities between these two methods and PINES that the authors should acknowledge and comment on. 

4. On page 13, lines17-38. The authors describe 639 annotations. Previously, the authors have convincingly argued that one of the key advantages of PINES over GWAVA, CADD is the cell type specific annotations. However, for DHS regions which is clearly cell type specific, only 1 value is included. This is rather puzzling.

5. On page 13, lines 35-37. Only 1 value for chromatin interactions is included. Again, such a phenomenon is cell type specific and also assay-specific. Why only 1 value is included? 

6. I am curious to know which of the 639 features are found to be informative in annotating these non-coding variants. The authors should comment on the different contributions of various annotations. 

7. As pointed out in the GWAVA paper (Ritchie et al. 2014), the epigenetics profile is very sensitive on the genomic context. Hence it is important to match the background variants with the variants supplied as input. For example, distance to the nearest TSS. If this is not consider, I suspect many false calls will be made. That is, non-functional variants will be selected for having a different epigenomic profile compared to the background SNPs. I think this needs to be checked. 

8. Page 6. Performance comparison on fine mapped GWAS risk loci. According to page 16, it seems that pre-computed scores were used for GWAVA, CADD, etc. This is okay but I think the authors should acknowledge that such a comparison is not exactly fair, since PINES is tuned using the input data. 

Minor: 
1. Page 14. Line 46. "The diagonal weight matrix W was constructed to assign weight 4 to one of the annotations and weight 1 to the remaining features." How are these values determined? Why 4 and 1? 
2. Page 7, line 42, "up-weighting of all cell types involved in immunity." Which cell types were considered immunity-involved?

**Reviewer 2:**

**Are the methods appropriate to the aims of the study, are they well described, and are necessary controls included?**
The methods are appropriate to the aims of the study. They are not well described. The controls that are included appear limited. The components of the process are not individually tested, making the overall value of work limited to a computational audience.

**Are the conclusions adequately supported by the data shown?**

The conclusions that are drawn are quite strong. It's worth noting that the most rigorous evaluation falls primarily to variants in 6 association peaks, and for at least one of these weights are manually applied.

**Are sufficient details provided to allow replication and comparison with related analyses that may have been performed?**

They are not, but I expect that with revisions that they could be.

**Is the method likely to be of broad utility? Is any software component easy to install and use?**

The software is not yet available for download on the website. No license in specified, so it is unclear how it could be reused if it was made available.

**Is the paper of broad interest to others in the field, or of outstanding interest to a broad audience of biologists?**

Yes: The question being addressed is important. The paper, as it stands, is not particularly important. However, it appears that there is potential that - with many revisions - it could be.

Comments to author:

The authors describe PINES, which is the name given to a relatively unsupervised method to predict the impact of noncoding variants. The precise sense in which the algorithm is unsupervised seems to depend on whether the weighted or unweighted form is used.

* The authors discuss PU learning, but there does not appear to be actual use of such methods. This should be cut or its relationship to PINES clearly specified.

* The algorithm appears to be quite simple: the authors appear to essentially count the number of independent annotations that a variant has accrued. However, the authors' description makes the algorithm seem highly complex. It's unclear if this is desired, but it substantially harms the readability of the manuscript. There appear to be no results that confirm a number of the assertions made with regards to the contributors to the algorithm's seeming success.

- The authors should compare the top 30 eigenvectors to other potential selections.

- The authors should compare to simple annotation counting.

* Because the approach relies so heavily on the proportion of the independent inputs that are annotated at the site, The algorithm may be exquisitely sensitive to the input data types. It would be helpful for the authors to incrementally add or remove annotation data to measure which data types (cell lines, annotation types) are most enhancing predictive performance in the current evaluations. It would also help to know whether the presence of numerous assayed immune cell types makes this method most well suited to the prediction of immune-related phenotypes. Of note, the analysis outside the immune-related set rests on only 6 variants.

* The primary evaluation appears to be across only 6 well-characterized variants. Thus some caution may be warranted in the broad utility of the method. However, the authors do not couch their findings with such caution. Also, only upon reading the methods section does it become clear that manual weighting is used for the pigmentation results in Figure 2. Was performance worse than other algorithms without manual weighting? The rationale, along with the use of manual weighting, needs to be clear to the reader within the results section.

* The authors claim that PINES is "Up to 78% better than other methods..." but certain other methods, such as GWAVA score very similarly to PINES. This framing should be adjusted as PINES should be compared to what scores as the second-best algorithm in the user's assessment.

* It's unclear to the extent that the user performing the evaluations was blinded to results when performing the "manual feature weighting" examples. This must be reported. If the user was not blinded, it would be helpful to provide some means to understand how confident readers should be that this result is not due to a search over weights to find those that performed well in this evaluation.

* No open source implementation appears to be available though it is asserted that some will be soon. This makes the code impossible to review. Given the growing focus on rigor, reproducibility, and transparency this lack appears to be hard to justify. Also, no license for the source code is specified. There are examples in the literature of software that has source released that is difficult or impossible to re-use due to the license. The choice of license should be considered when determining the likely impact of the work.

* How the enrichment p-values that are used in the weighted approach are generated is unspecified. What test is employed?

* The authors thank Dr. Alex Bloemendal for coming up with the use of a low-rank approximation of the annotation covariance matrix. This appears to be one of the primary contributions to the overall work, as without this the work would appear to be largely equivalent to annotation counting. At the very least, assessment should be performed with and without this step to determine its impact. If this is critical to the performance of the method, one may wonder whether an acknowledgement alone is sufficient.

**Reviewer 3:**

**Are the methods appropriate to the aims of the study, are they well described, and are necessary controls included?**
The methods are not described in sufficient clarity/detail

**Are the conclusions adequately supported by the data shown?**

Partially, see below.

**Are sufficient details provided to allow replication and comparison with related analyses that may have been performed?**

No

**Is the method likely to be of broad utility? Is any software component easy to install and use?**

Yes

**Is the paper of broad interest to others in the field, or of outstanding interest to a broad audience of biologists?**

Yes: An interesting new computational method

Comments to author:

Bodea and colleagues present a manuscript about how phenotype-specific information improves the prediction of functional

impact for noncoding variants.

Comments

The method appears clever and the comparison the authors present shows that it performs well.

The authors appear not to have done their due diligence to identify other competing methods. It is hard to make this comment without revealing my identity, and so I will just say that we have published a method that uses phenotype to drive the identification of non-coding Mendelian variants:

Smedley D, Schubach M, Jacobsen JOB, Köhler S, Zemojtel T, Spielmann M, Jäger

M, Hochheiser H, Washington NL, McMurry JA, Haendel MA, Mungall CJ, Lewis SE,

Groza T, Valentini G, Robinson PN. A Whole-Genome Analysis Framework for

Effective Identification of Pathogenic Regulatory Variants in Mendelian Disease.

Am J Hum Genet. 2016 Sep 1;99(3):595-606.

we have also published a fuller description of the method and show that it has a superior performance to the competing methods described in the current manuscript.

Schubach M, Re M, Robinson PN, Valentini G. Imbalance-Aware Machine Learning

for Predicting Rare and Common Disease-Associated Non-Coding Variants. Sci Rep.

2017 Jun 7;7(1):2959. doi: 10.1038/s41598-017-03011-5. PubMed PMID: 28592878;

PubMed Central PMCID: PMC5462751.

The authors should ideally compare their method to ours, or at least discuss why they do not think a comparison is appropriate.

The methods description is difficult to follow because of many statements such as "a list of CAGE enhancers was used to create Fig 5" etc. However, if we go to the legend of Figure 5, there is not really enough information to follow the method. For instance, how were the FANTOM enhancers chosen? How were the tissue specific variants chosen? etc.

I think the methods would be easier to follow if the authors described them step by step.

I think it is also important that a bioinformatician should be able to understand the algorithms used in the paper without having to examine the source code. Statements like this in the methods are extremely imprecise: "Noncoding background variants were selected randomly across the genome, and we used the ClinVar database [41] and GWAS Catalog [42] to ensure that no overlap exists with known functional loci." There are many ways of selecting "background" variants randomly. In my opinion, the concept of "background variant" is not well defined anyway. The authors do not say how much overlap they tolerate, and do not say which variants they took from ClinVar (benign or pathogenic etc., and if they also include variants with conflicting evidence etc etc).

(Note further below the authors say they randomly chose 150000 common variants, but still they should state their criteria for common and for random. Given the multitude of known and unknown correlations across the genome, the authors need to make more clear their strategy for avoiding overfitting by an apprpriate choice of training and testing variants -- I did not see enough if this in the manuscript).

The description of the actual PINES method is clearer, but again the authors could go into more detail. The chose the top 30 eigenvectors based on a scree plot of a PCA of the covariance matrix, but they could also state how much total variance is covered etc.

The authors state that the all-1 annotation vector 1 is significant since it provides the direction of a point with

maximal annotation load, and thus the greatest evidence for functionality. This assertion is not critical for understanding the method, but I think it is wrong -- it is mixing up the evidence of some biochemical mark such as a chromatin mark with biologically or medically relevant function (this was the same "controversy" surrounding claims that ENCODE showed that 80% of the genome has a function, which can be found by googling). The same comments applies further down to the claim "Smaller scores indicate greater evidence for functionality." I believe the authors should be more cautious with their interpretation of the data and avoid unsubstantiated statements about biological functionality -- I agree with the authors that this is plausible but there is no need to use language that is "hyped" to describe this nice method.

"Following the approach presented in [47], we additionally scale this angle by the length of the projected x vector,"

=> Again it would be helpful for the reader to summarize in one sentence the approach in 47 and to motivate why it is being used in PINES.

I would also like to see the source code and the data available to the reviewers -- the authors say it will be made available later.

The authors do not comment on the fact that an unknown proportion of the GWAS hits is likely to be functional with the rest being Tag SNPs. Since their method is posited upon being able to use functional data to prioritize variants, this needs to be discussed more. I am surprised that the authors did not attempt to test their method on some subset of GWAS hits that have been characterized as "functional" by some other method -- would this be a good idea? Can the authors show data on this?

**Authors’ response to reviews**

We thank the reviewers for their comments, which we believe led to a significantly improved manuscript. We respond to each of the reviewers' comments below.

Reviewer #1: Bodea et al. describe the computational framework PINES (Phenotype-Informed Noncoding Element Scoring) which evaluates the functional impact of noncoding variants in an unsupervised setting by integrating epigenetic annotations from diverse sources in a phenotype dependent manner. The authors showed PINES enjoys superior performance when comparing to competing methods such as CADD. PINES can also help interpret the results of finding mapping studies and predict novel causal alleles of complex diseases. Here are my comments.

1. Overall, I feel PINES has interesting ideas that differ from most of existing methods. It focuses on noncoding variants, unsupervised, and uses cell type specific features. On the other hand, all of these have been implemented in one of more existing methods, albeit not in the same one. Since only a limited number of phenotypes is tested, it is unclear how significant is this new method.

*We agree with the reviewer that PINES is unique in bringing together several approaches already available in other tools, thus increasing user friendliness over previous methods. Beyond that, the novel statistical framework behind PINES adds improved power to detect functional noncoding variants impacting both Mendelian and complex phenotypes.*

*We disagree however that PINES has been tested only on a limited number of phenotypes. In our manuscript we assess the performance of PINES across multiple immune, neurologic, cardiometabolic, and developmental phenotypes, as well as on variants reported as impacting intermediary phenotypes (MPRA and FANTOM5 data). In addition, we have run unweighted PINES on a collection of noncoding variants used by ReMM (Smedely D. et al. Am. J. Hum. Genet. 2016) as training set, as well as a collection of GWAS variants used by hyperSMURF (Schubach M. et al. Sci. Rep. 2017) as training set. Both variant sets have been linked to a wide range of phenotypes. Even when run in unweighted mode, PINES achieved excellent classification performance (AUROC = 0.87 for the Mendelian set and AUROC = 0.91 for the GWAS set). Our results show that PINES is widely applicable across phenotypes and can score both high-penetrance Mendelian variants as well as more common GWAS risk variants.*

2. The authors claim that PINES is "unsupervised". But they state that "A unique feature of PINES is that analyses may be customized towards genomic annotations of the highest relevance to phenotypes of interest." Such annotations include GWAS-implicated region to serve as positive control. I feel that these two statements contradict each other. In fact, the "weighted" version of PINES is clearly supervised.

*PINES run in either unweighted mode or in manual weighted mode is unsupervised: no examples of functional or non-functional variants are required, and the scoring is performed solely based on the available annotations and their properties (correlation structure, pre-specified weights). In our revised manuscript, we have rewritten the "Choice of feature weights" section under Methods to include detailed explanations that now should better guide the user when choosing a weighted mode. We have also added the following paragraph to the Introduction to describe the different PINES scoring schemes, and to clarify how user-specified variants that are phenotypically relevant are used by PINES:*

*"Here we introduce the Phenotype-Informed Noncoding Element Scoring (PINES) framework. PINES enables a phenotype-dependent scoring of noncoding variants that can be customized towards annotations considered as of highest relevance to a phenotype of interest. During the PINES scoring process, such annotations are assigned the highest weights, while annotations of lower relevance to a phenotype are down-weighted. Moreover, other than most previous scoring tools, PINES uses an unsupervised approach to systematically assess the functional significance of noncoding SNVs and indels. In particular, no variant sets are used to train the PINES model in the unweighted and manually weighted settings. When no phenotype-relevant information is available, PINES scores individual variants solely by comparing them to the background level of annotations across the genome, while taking the correlation structure between annotations into account. When the user specifies individual annotations as phenotype-relevant, this information is directly reflected in the annotation weights. PINES can also automatically determine weights if the user specified a set of variants that are relevant for the phenotype of interest (for example GWAS lead SNPs). Under this semi-supervised setting, the specified variants are only used to aid in the selection of annotation weights: after an enrichment analysis is performed independently for each annotation (see "Choice of feature weights" section under Methods) no further use is made of the variants, and the variants are never processed through the full PINES framework or scored."*

3. There are a couple of recent studies, namely GenoCanyon, a whole-genome functional annotation approach based on unsupervised statistical learning (Lu et al. Scientific Reports 2015); and DIVAN, a disease-specific, non-coding variant annotation tool (Chen et al. Genome Biology 2016). I feel that there are some similarities between these two methods and PINES that the authors should acknowledge and comment on.

*Thank you for pointing out these two new methods. We have updated Figures 3 and 4 to include comparisons to GenoCanyon and DIVAN. PINES run in weighted annotation mode outperforms both GenoCanyon and DIVAN for the variants and phenotypes tested, as reflected by lower p-values and higher AUROC scores. In addition, while DIVAN has released precomputed scores for 45 diseases, PINES can be queried with minimal computational overhead to provide custom scores for many combinations of biologically relevant annotations.*

4. On page 13, lines17-38. The authors describe 639 annotations. Previously, the authors have convincingly argued that one of the key advantages of PINES over GWAVA, CADD is the cell type specific annotations. However, for DHS regions which is clearly cell type specific, only 1 value is included. This is rather puzzling.

*DHS annotations are included individually for each of the 127 epigenomes (first bullet point). In addition, the DHS regions mentioned in the third bullet point represent ChrommHMM predictions that aggregate annotation across all cell types. To clarify, we have rephrased this section in the manuscript as such:*

*"Presence or absence of a DHS region as predicted by the ChromHMM 15 state model trained  on all epigenomes (1 value). This annotation represents ChrommHMM predictions that aggregate annotation across all cell types, so only one value is provided. However, cell-type specific DHS data is also included (see the first bullet point)."*

5. On page 13, lines 35-37. Only 1 value for chromatin interactions is included. Again, such a phenomenon is cell type specific and also assay-specific. Why only 1 value is included?

*This is a good observation: since the 4DGenome database is based on literature curation, there is substantial variability between the number of reported chromatin interactions for different cell types (Jurkat cells have only 6 entries in the database, while IMR-90 cells have over 1.2 million entries). Due to this issue, we decided to aggregate the available chromatin interactions across cell types, and treat this as a non-tissue-specific annotation. To clarify, we have rephrased this section in the manuscript as such:*

*"Presence or absence of a region involved in chromatin interactions with other regions as reported in the 4DGenome database (1 value). Due to the substantial variability between the number of chromatin interactions reported for different cell types in the 4DGenome database, we aggregate the available chromatin interaction data across all cell types and treat this as one non-tissue-specific annotation."*

6. I am curious to know which of the 639 features are found to be informative in annotating these non-coding variants. The authors should comment on the different contributions of various annotations.

*In the unweighted use case, an annotation that is independent of all other annotations will contribute to the distances in the de-correlated space more than an annotation that is part of a correlated group. Thus, having multiple uncorrelated annotations will result in a more significant PINES p-value than having the same number of correlated annotations (see our answer to minor comment #1).*

*In the context of a given phenotype, PINES can determine annotation weights based on user-specified variants that are relevant to the phenotype (for example GWAS SNPs):*

*●	Figure S8 highlights the weights placed by PINES on cell-type specific annotations when IBD GWAS lead SNPs are provided as input by the user. To note is the high weight placed on immune and GI cells, two distinct cell groups that are known to be involved in IBD pathology.*

*●	Our new Figure S9 shows the weights placed by PINES on cell-type specific annotations when MS GWAS lead SNPs are provided as input by the user. To note is the high weight placed on immune cells, which is expected given the autoimmune etiology of MS. PINES also upweights annotations related to the thymus, which has been connected to MS pathology as a source of autoreactive T-cells.*

*We have added a paragraph to the "Choice of feature weights" section under Methods describing the results of Figures S8 and S9. This section also contains an updated discussion on choosing manual weighting constants, which is relevant to minor comment #1.*

7. As pointed out in the GWAVA paper (Ritchie et al. 2014), the epigenetics profile is very sensitive on the genomic context. Hence it is important to match the background variants with the variants supplied as input. For example, distance to the nearest TSS. If this is not consider, I suspect many false calls will be made. That is, non-functional variants will be selected for having a different epigenomic profile compared to the background SNPs. I think this needs to be checked.

*As Figure 1 in the Ritchie et al. 2014 paper highlights, the choice of background variants can have a significant impact on AUROC values: a more stringent matching delivers fewer positives, but will also yield many false negatives. Since it is currently unclear which matching algorithm is optimal for a given locus and phenotype, one approach is to provide multiple scores for every SNP based on different background variants (this is the approach GWAVA follows). We would like to point out a few caveats of this approach:*

*●	GWAVA exhibits loss of power under TSS and region matching. For example, throughout our manuscript we have used "unmatched" GWAVA scores in our comparisons (i.e. the background is selected from across the genome), since we noticed that using GWAVA scores based on more stringent background matching leads to reduced signal.*

*●	Providing multiple scores for each variant to users can complicate interpretation of the results. For example, if a variant achieves a given significance threshold under genome-wide matching but not under TSS-based matching, it is unclear whether it should be discarded from further consideration.*

*Given these concerns, PINES addresses the issue of background set selection in two ways:*

*●	Our background set is representative of a wide range of TSS distances:*

*○	first 25% of variants: 0 kb - 25kb from the nearest TSS*

*○	second 25% of variants: 25kb - 87kb from the nearest TSS*

*○	third 25% of variants: 87kb - 256kb from the nearest TSS*

*○	fourth 25% of variants: more than 256kb from the nearest TSS*

*●	In Figure 2 we demonstrate that inspection of a regional PINES score plot can point out the most likely functional variant against the local background, without the need to compute region-matched scores.*

*Finally, we investigated whether PINES scores are robust to the selection of background variants by re-computing unweighted PINES p-values for a set of 50 variants based on repeated random draws of background sets (background set size = 10,000 variants). The resulting p-values were highly correlated (minimum pairwise correlation = 0.986), indicating that the current selection of background variants is representative for the distribution of epigenetic annotations across the genome.*

*We have added a detailed description of the background variant selection process, including the distribution of distances to the nearest TSS, in the "Background variants and the null distribution" section under Methods.*

8. Page 6. Performance comparison on fine mapped GWAS risk loci. According to page 16, it seems that pre-computed scores were used for GWAVA, CADD, etc. This is okay but I think the authors should acknowledge that such a comparison is not exactly fair, since PINES is tuned using the input data.

*We would like to clarify that PINES is not tuned using the input (fine-mapped variant sets). The original GWAS hits are used to highlight general phenotypic relevant annotations, and then any noncoding variant across the genome can be scored based on this information. Since the other methods are not able to account for such phenotype-specific input, and users would likely resort to the published pre-computed scores due to their ease of use, we believe that our comparison to weighted PINES scores is an appropriate way to showcase the benefits of our approach.*

Minor:

1. Page 14. Line 46. "The diagonal weight matrix W was constructed to assign weight 4 to one of the annotations and weight 1 to the remaining features." How are these values determined? Why 4 and 1?

*Unweighted PINES scores are computed by setting the weight matrix W to the identity matrix (all weights = 1). The problem of up-weighting a subset of annotations is equivalent to specifying a constant to serve as weight for the annotations of interest. If this constant is set to 1, then we are in the unweighted case. If the constant is greater than 1, then more weight is placed on the corresponding annotations.*

*To determine an appropriate value for the weighting constant, it is important to note that increasing the value beyond a certain threshold results in two outcomes:*

*1.	the increase will stop adding power to detect variants overlapping the annotations of interest*

*2.	the increase will significantly reduce the power to detect variants overlapping other annotations*

*An appropriate weighting constant will thus provide sufficient power to detect variants overlapping annotations of interest, while still allowing PINES to detect signal that does not stem from the upweighted annotations. While the exact choice of weighting constant will thus depend on the upweighted annotations and their correlation structure, we have empirically observed via simulations that a weighting constant of 4 generally achieves a good balance of power given the correlations present in the ENCODE and Roadmap data.*

*Our new Figure S13 formalizes this argument by highlighting the dynamics of weighted PINES scores in a simulated dataset as the weighting constant is increased. For this analysis we generate ten sets of correlated annotations. Each set contains ten variants, and no correlation exists between variants of different sets (panel a). We then select the first annotation block as phenotypically relevant, and assign the same weighting constant to all ten annotations within the block. This simulates the presence of phenotypically-relevant annotations that the user upweights. We compute the PINES significance level for two variants as the weighting constant is increased from 0 to 20 in 0.5 increments:*

*●	Variant 1: overlaps all ten upweighted annotations and none of the remaining 90 annotations (panel b)*

*●	Variant 2: overlaps none of the ten upweighted annotations and all of the remaining 90 annotations (panel c)*

*As the weighting constant increases, we observe that the weighted PINES p-value decreases for variant 1 and increases for variant 2. This depicts the tradeoff in power that can be used to select an appropriate weighting constant.*

*We have significantly rewritten the "Choice of feature weights" section under Methods, which now also includes a clarification of this point.*

2. Page 7, line 42, "up-weighting of all cell types involved in immunity." Which cell types were considered immunity-involved?

*We upweighted all annotations related to primary T cells and primary B cells. We have added the following clarification to the highlighted section:*

*"The panel highlighting all immune-related candidate variants groups together variants across many immune disorders, and thus relies on a uniform manual up-weighting of all cell types involved in immunity (primary T and B cells). Classification accuracy is increased in this setting as well compared to an unweighted analysis, but an enrichment-based weighting of features is preferred."*

Reviewer #2: The authors describe PINES, which is the name given to a relatively unsupervised method to predict the impact of noncoding variants. The precise sense in which the algorithm is unsupervised seems to depend on whether the weighted or unweighted form is used.

* The authors discuss PU learning, but there does not appear to be actual use of such methods. This should be cut or its relationship to PINES clearly specified.

*We have removed the reference to PU learning.*

* The algorithm appears to be quite simple: the authors appear to essentially count the number of independent annotations that a variant has accrued. However, the authors' description makes the algorithm seem highly complex. It's unclear if this is desired, but it substantially harms the readability of the manuscript. There appear to be no results that confirm a number of the assertions made with regards to the contributors to the algorithm's seeming success.

- The authors should compare the top 30 eigenvectors to other potential selections.

- The authors should compare to simple annotation counting.

*We have added a new section under Methods ("Comparison of PINES and annotation counting") that presents a detailed comparison of the two approaches. The results highlight that PINES, as opposed to annotation counting, appropriately models the correlation structure of the annotation data. The PINES methodology thus provides scores that can diverge strongly from annotation counting. This point is particularly important given the known correlation structure present in the ENCODE and Roadmap Epigenomics datasets. Additionally, PINES is able to modulate the importance of individual annotations in the scoring procedure, while at the same time taking the correlation structure into account. Annotation counting would rely on subsetting the annotation database, thus rendering potentially informative annotations (annotations correlated to the annotations of interest) invisible to the scoring procedure.*

*To formalize this argument, we have now added an analysis of PINES relative to annotation counting as a new Figure S12 and provide details on this analysis in the Methods section. Specifically, we simulate annotations under two scenarios:*

*1.	No correlation structure is present in the annotation space (panel a). We generate 10,000 variants with 100 independent binary annotations (0/1).*

*2.	Annotations exhibit correlation structure (panel d). Here we generate 10,000 variants with 10 annotation blocks, each block containing 10 binary annotations (0/1). The annotations within each block are correlated, but no correlation exists between annotations in different blocks.*

*We highlight two variants:*

*1.	The variant that overlaps the first annotation from each of the 10 blocks of correlated annotations (green).*

*2.	The variant that overlaps all ten annotations from the first block of correlated annotations (blue).*

*Thus, both the green and the blue variant overlap ten annotations, but the selected annotations reflect different correlation patterns (uncorrelated annotations for green, correlated annotations for blue).*

*We next designate the first annotation from the first correlation block as biologically relevant, and use this selection to explore the effect of weighting on PINES scores. In particular, both the green and the blue variants overlap the biologically relevant annotation. Panels c and f plot weighted PINES scores, where all variants that do not overlap the upweighted annotations are marked black.*

*Figure S12 highlights two points:*

*1.	As opposed to raw counts, PINES scores are sensitive to correlation structure in the annotation space. Panel b shows almost equal PINES scores for the green and blue variant when no correlation structure is present in the annotation data. Under this scenario, PINES p-values and raw annotation counts will provide a similar ranking of the variants. Panel c shows that variants overlapping the upweighted annotation achieve more significant p-values than variants that do not overlap this annotation. When correlation structure is present, panel e shows the PINES and annotation counting deliver different results: the variant that overlaps a block of correlated annotations (blue) achieves less significant PINES p-values than the variants that overlaps multiple independent annotations (green). This distinction is not reflected by annotation counts, where both variants overlap a total of ten annotations. Panel f shows that correlation structure is also properly accounted for by weighted PINES scores.*

2.	As opposed to raw counts, PINES offers a principled method to perform annotation weighting. Panels c and f highlight the effect of annotation weights on PINES p-values under two different correlation structures of the annotation data.

*We have included these points in the new "Comparison of PINES and annotation counting" section under Methods.*

* Because the approach relies so heavily on the proportion of the independent inputs that are annotated at the site, The algorithm may be exquisitely sensitive to the input data types. It would be helpful for the authors to incrementally add or remove annotation data to measure which data types (cell lines, annotation types) are most enhancing predictive performance in the current evaluations. It would also help to know whether the presence of numerous assayed immune cell types makes this method most well suited to the prediction of immune-related phenotypes. Of note, the analysis outside the immune-related set rests on only 6 variants.

*The reviewer mentions that "the analysis outside the immune-related set rests on only 6 variants". We disagree with this statement, and aim to clarify the scope of our analyses in the paragraphs below.*

*Although numerous immune cell types are represented in the Roadmap and ENCODE datasets, we directly show that PINES is well suited for the analysis of non-immune phenotypes:*

*●	Figures 2 and 3 present results for schizophrenia, Parkinson's disease, blood lipids, pigmentation, cleft lip with or without cleft palate, and QRS prolongation.*

*●	Figure 4 presents results for functional variants that have only been connected to intermediary phenotypes (MPRA, FANTOM5).*

*●	We have additionally tested the performance of unweighted PINES to detect signal on the training set of rare Mendelian variants used by ReMM (Smedely D. et al. Am. J. Hum. Genet. 2016) and hyperSMURF (Schubach M. et al. Sci. Rep. 2017) (AUROC = 0.87), as well as for the GWAS variants used as training set by hyperSMURF (AUROC = 0.91). PINES thus shows good classifier accuracy on both of these variant sets, covering a wide range of Mendelian and complex phenotypes.*

*Given the good performance of PINES across a range of phenotypes and disease architectures, we conclude that the current annotation set offers sufficient diversity of cell types and tissues for the majority of use cases. However, we agree with the reviewer that future improvements in the quality and breadth of epigenetic reference datasets (additional cell types, additional assays) will also improve the power of functional prediction methods in general. For example, expanding annotation datasets with additional kidney cell types will improve functionality predictions for kidney disease risk variants.*

* The primary evaluation appears to be across only 6 well-characterized variants. Thus some caution may be warranted in the broad utility of the method. However, the authors do not couch their findings with such caution. Also, only upon reading the methods section does it become clear that manual weighting is used for the pigmentation results in Figure 2. Was performance worse than other algorithms without manual weighting? The rationale, along with the use of manual weighting, needs to be clear to the reader within the results section.

*We disagree with the reviewer's comment that the analysis presented in Figure 2 is our primary evaluation. Our answer to the previous comment should help to clarify this point. However, it is worth underlining that the 6 variants the reviewer refers to are part of a still very small number of well-characterized (through experiments and patient studies), highly-penetrant non-coding SNPs. We considered these variants as the optimal set for the initial benchmarking of the dynamic range of PINES. We then expanded this benchmarking into a large collection of fine-mapped variants, and finally to variants residing in FANTOM5 enhancers and identified as MPRA hits.*

*Weighted PINES scores were used in all examples in Figure 2. Manual weighting (as opposed to weighting based on GWAS peak enrichments) was applied to the pigmentation variants since, to our knowledge, no GWAS of pigmentation exists that would identified enough signals for a well-powered enrichment analysis. We have clarified this point in the Results section (first paragraph of the section titled "PINES prioritizes causal noncoding variants with high confidence"):*

*"Weighted PINES was used when genome-wide significant SNPs were identified by GWAS for the trait under consideration, and manual weighting was used for the pigmentation loci since, to our knowledge, no GWAS of pigmentation exists to date that has identified enough peaks for a well-powered enrichment analysis (see Methods)."*

* The authors claim that PINES is "Up to 78% better than other methods..." but certain other methods, such as GWAVA score very similarly to PINES. This framing should be adjusted as PINES should be compared to what scores as the second-best algorithm in the user's assessment.

*We have updated our description of the results and now more carefully describe the performance of PINES relative to the other tools. It is worthwhile to note that no single algorithm consistently scored second-best in our comparisons, indicating a considerable variability of the other tools' performance across datasets.*

* It's unclear to the extent that the user performing the evaluations was blinded to results when performing the "manual feature weighting" examples. This must be reported. If the user was not blinded, it would be helpful to provide some means to understand how confident readers should be that this result is not due to a search over weights to find those that performed well in this evaluation.

*Throughout the manuscript, all examples that employ manual feature weighting use a weighting constant of 4. This constant was chosen based on generic simulation results that we describe in our updated "Choice of feature weights" section under Methods. No optimization of this constant was performed to improve the performance of PINES for specific phenotypes. As a follow-up to the reviewer's comment we have re-computed PINES scores across the pigmentation loci in Figure 2 and have actually found that a constant of 2 would improve the significance levels of the causal variants even further.*

*To provide more detail on the behavior of PINES scores under different weighting constants, we discuss manual weighting below (and even more detail is available in the updated "Choice of feature weights" section under Methods). In short, we highlight the following:*

*●	The choice of manual weighting constant is affected by the selection of biologically relevant annotations and their correlation structure, so no single value will perform equally well under all scenarios.*

*●	There is a predictable trend in weighted PINES scores as this constant varies, which can serve as a guide to the user.*

*●	Increasing the constant beyond a threshold adversely affects the power of weighted PINES, so this feature cannot be misused to boost variants that have little overlap with biologically relevant annotations.*

*●	The ability to perform a search over weights is a useful tool for PINES users, since the change in weighted PINES scores can pinpoint variants that have strong overlap with the selected annotations. For example in a study of IBD where both immune and GI pathologies are present, users are able to determine which variants are likely to drive the immune axis, and which variants are likely to drive the GI axis.*

*Unweighted PINES scores are computed by setting the weight matrix W to the identity matrix (all weights = 1). The problem of up-weighting a subset of annotations is equivalent to specifying a constant to serve as weight for the annotations of interest. If this constant is set to 1, we are in to the unweighted case. If the constant is greater than 1, then more weight is placed on the corresponding annotations.*

*To determine an appropriate value for the weighting constant, it is important to note that increasing the value beyond a certain threshold results in two outcomes:*

*●	the increase will stop adding power to detect variants overlapping the annotations of interest*

*●	the increase will significantly reduce the power to detect variants overlapping other annotations*

*An appropriate weighting constant will provide sufficient power to detect variants overlapping annotations of interest while still allowing PINES to detect signal even if it does not stem from the upweighted annotations.*

*Figure S13 formalizes this argument by highlighting the dynamics of weighted PINES scores in simulated data as the weighting constant increases. For this analysis we generate ten sets of correlated annotations. Each set contains ten binary annotations (0/1), and no correlation exists between annotations of different sets (panel a). We then select the first block of annotations as biologically relevant, and assign the same weighting constant to all ten annotations within the block. This simulates the presence of phenotypically-relevant annotations that the user upweights. We compute the PINES significance level for two variants as the weighting constant increases from 0 to 20 in 0.5 increments:*

*●	Variant 1: overlaps all ten upweighted annotations and none of the remaining 90 annotations (Panel b)*

*●	Variant 2: overlaps none of the ten upweighted annotations and all of the remaining 90 annotations (Panel c)*

*As the weighting constant increases, we observe that the weighted PINES p-value decreases for variant 1 and increases for variant 2. This depicts the tradeoff in power that can be used to select an appropriate weighting constant.*

*In conclusion, a search over weights is a useful tool for the user, since it has the potential to highlight variants that overlap annotations of interest. Once a weight has been found that provides sufficient power to detect relevant variants, further increasing the weight will hurt the performance of PINES. Also, since the p-values in panel c are strictly increasing, it is not possible to promote a variant that lacks overlap with the upweighted annotations just by increasing the weighting constant.*

*We have significantly expanded the "Choice of feature weights" section in Methods to clarify this point.*

* No open source implementation appears to be available though it is asserted that some will be soon. This makes the code impossible to review. Given the growing focus on rigor, reproducibility, and transparency this lack appears to be hard to justify. Also, no license for the source code is specified. There are examples in the literature of software that has source released that is difficult or impossible to re-use due to the license. The choice of license should be considered when determining the likely impact of the work.

*The source code and all annotations are now available through the PINES website (http://genetics.bwh.harvard.edu/pines/) under the GNU General Public License.*

* How the enrichment p-values that are used in the weighted approach are generated is unspecified. What test is employed?

*Enrichments are computed by performing 1000 draws of variant sets that are matched by allele frequency to the user-specified input variants (for example GWAS lead SNPs). We then compare the total number of GWAS variants overlapping each annotation with the number of overlaps in the matched sets to compute an empirical enrichment p-value. Using IBD and MS as examples, we show in Figures S8 and S9 that the enrichments computed via this approach prioritize tissues with known involvement in disease processes.*

*We have included a clarification of our approach in the "Choice of feature weights" section under Methods.*

* The authors thank Dr. Alex Bloemendal for coming up with the use of a low-rank approximation of the annotation covariance matrix. This appears to be one of the primary contributions to the overall work, as without this the work would appear to be largely equivalent to annotation counting. At the very least, assessment should be performed with and without this step to determine its impact. If this is critical to the performance of the method, one may wonder whether an acknowledgement alone is sufficient.

*We have performed an in-depth comparison of PINES and annotation counting to answer one of the reviewer's previous questions. We wish to reiterate that PINES appropriately models the correlation structure of the annotation data, and offers a well-powered approach to annotation weighting. In contrast, annotation counting does not address these points, and can deliver spurious results when correlation structure is present.*

*We have also evaluated whether the low-rank approximation significantly improves the performance of PINES. Comparing PINES scores with and without the approximation on a set of 20,000 background variants suggests that this additional processing of the annotation covariance matrix has no significant impact on the results.*

*However, since the advice to implement a low-rank approximation step does have statistical validity and can prove useful as the PINES annotation database continues to grow, we have included Dr. Bloemendal as a co-author on this manuscript.*

Reviewer #3: Bodea and colleagues present a manuscript about how phenotype-specific information improves the prediction of functional

impact for noncoding variants.

Comments

The method appears clever and the comparison the authors present shows that it performs well.

The authors appear not to have done their due diligence to identify other competing methods. It is hard to make this comment without revealing my identity, and so I will just say that we have published a method that uses phenotype to drive the identification of non-coding Mendelian variants:

Smedley D, Schubach M, Jacobsen JOB, Köhler S, Zemojtel T, Spielmann M, Jäger

M, Hochheiser H, Washington NL, McMurry JA, Haendel MA, Mungall CJ, Lewis SE,

Groza T, Valentini G, Robinson PN. A Whole-Genome Analysis Framework for

Effective Identification of Pathogenic Regulatory Variants in Mendelian Disease.

Am J Hum Genet. 2016 Sep 1;99(3):595-606.

we have also published a fuller description of the method and show that it has a superior performance to the competing methods described in the current manuscript.

Schubach M, Re M, Robinson PN, Valentini G. Imbalance-Aware Machine Learning

for Predicting Rare and Common Disease-Associated Non-Coding Variants. Sci Rep.

2017 Jun 7;7(1):2959. doi: 10.1038/s41598-017-03011-5. PubMed PMID: 28592878;

PubMed Central PMCID: PMC5462751.

The authors should ideally compare their method to ours, or at least discuss why they do not think a comparison is appropriate.

*We thank the reviewer for pointing out these methods (which we now mention in the Introduction and have added to the References). It is important to note that a few significant differences exist between PINES and ReMM or hyperSMURF:*

*●	Unlike PINES, ReMM and hyperSMURF do not perform annotation-specific analyses directly. ReMM is part of the Genomiser framework that includes phenotypic similarity as a separate component into the workflow.*

*●	ReMM and hyperSMURF have been carefully designed to address issues arising from the imbalance between available negative and positive training data, i.e., they are supervised. Since PINES does not make use of training sets of functional or non-functional noncoding variants, it is not affected by such imbalances.*

*●	Based on a manually curated training set of Mendelian disease-associated variants, ReMM is aimed at prioritizing noncoding variants in Mendelian disease. hyperSMURF can be applied to variants involved in Mendelian and complex disease, but a different training set is used in each scenario. We show that PINES scores can be used broadly across different phenotypes, as well as different disease architectures, with good performance.*

*Given these differences we envision weighted PINES scores as complementary to ReMM or hyperSMURF in a Genomiser setting, and consider that a direct comparison would be difficult to interpret.*

*The methods description is difficult to follow because of many statements such as "a list of CAGE enhancers was used to create Fig 5" etc. However, if we go to the legend of Figure 5, there is not really enough information to follow the method. For instance, how were the FANTOM enhancers chosen? How were the tissue specific variants chosen? etc.*

*We first fixed a typo in the paragraph highlighted by the reviewer: the reference should be to Figure 4. Since no phenotype has been recorded for the variants residing in FANTOM5 enhancers or for the MPRA hits, we only performed an unweighted PINES analysis. Thus no tissue-specific information was included in the scoring process. We used all CAGE-based human enhancers reported by FANTOM5 (phases 1 and 2 combined). When defining our initial set of background variants, we found 9000 variants that overlapped with these enhancer regions (see also our answer to the question below on background variant selection). We excluded these variants from the background set, and instead scored them via PINES to investigate whether any enrichment in epigenetic signal can be detected relative to the genome-wide background.*

*To clarify this point in the manuscript, we have added the following paragraph in the "Unweighted PINES scores detect signal at expression-modulating variants identified in a multiplexed reporter assay, and for variants residing in FANTOM5 CAGE-defined enhancers" section under Results:*

*"We next compared the power of PINES, Eigen-PC, CADD, GWAVA, DANN, LINSIGHT, GenoCanyon, and FATHMM-MKL to correctly prioritize 9,000 variants residing within enhancers that have been identified by the FANTOM5 project through cap analysis of gene expression (CAGE) [6]. Since no phenotype has been recorded for the variants residing in FANTOM5 enhancers or for the MPRA hits, we only performed an unweighted PINES analysis. Thus no tissue-specific information was included in the scoring process. We used all CAGE-based human enhancers reported by FANTOM5 (phases 1 and 2 combined). When defining our set of background variants, we found 9000 variants that overlapped with these enhancer regions (see section "Background variants and the null distribution" under Methods). We excluded these variants from the background set, and instead scored them via PINES to investigate whether any enrichment in epigenetic signal can be detected relative to the genome-wide background. PINES correctly assigned noncoding variants residing in these regions the highest relevance scores relative to genomic background variation (Fig. 4)."*

I think the methods would be easier to follow if the authors described them step by step. I think it is also important that a bioinformatician should be able to understand the algorithms used in the paper without having to examine the source code. Statements like this in the methods are extremely imprecise: "Noncoding background variants were selected randomly across the genome, and we used the ClinVar database [41] and GWAS Catalog [42] to ensure that no overlap exists with known functional loci." There are many ways of selecting "background" variants randomly. In my opinion, the concept of "background variant" is not well defined anyway. The authors do not say how much overlap they tolerate, and do not say which variants they took from ClinVar (benign or pathogenic etc., and if they also include variants with conflicting evidence etc etc).

(Note further below the authors say they randomly chose 150000 common variants, but still they should state their criteria for common and for random. Given the multitude of known and unknown correlations across the genome, the authors need to make more clear their strategy for avoiding overfitting by an apprpriate choice of training and testing variants -- I did not see enough if this in the manuscript).

*To provide an overview of the PINES algorithm, we present a step-by-step workflow in Figure 1. We have also added more in-depth descriptions (including significantly updated sections on annotation weights and background variants, and a new section comparing the performance of PINES vs. annotation counting) throughout the Results and Methods sections.*

*Regarding the selection of background variants, we performed the following steps:*

*1.	Extract a random subset of 200,000 rs IDs from dbSNP build 137. No additional criteria were used in the randomization process at this stage.*

*2.	Annotate the resulting variant set with ClinVar data, and remove all "Pathogenic", "Likely pathogenic", and "Uncertain significance" variants.*

*3.	From the resulting variant set, remove all variants that are present in the GWAS Catalog, or overlap FANTOM5 CAGE-based human enhancer regions.*

*4.	Annotate the remaining variants with 1000 Genomes continental allele frequencies (AFR, AMR, ASN, EUR), and remove all variants with frequency < 5% for any of the continental ancestries.*

*This stepwise filtering results in a background set of 155,000 variants. The median distance between two consecutive background SNPs is 5.7kb, which indicates a good coverage of the entire genome. We observe only 4,387 background variants (about 2.8% of the background set) that are in LD (r^2 > 0.8) with any GWAS Catalog variant. Our background set is also representative of a wide range of distances to the nearest transcription start site (TSS):*

*●	first 25% of variants: 0 kb - 25kb from the nearest TSS*

*●	second 25% of variants: 25kb - 87kb from the nearest TSS*

*●	third 25% of variants: 87kb - 256kb from the nearest TSS*

*●	fourth 25% of variants: more than 256kb from the nearest TSS*

*Since regions close to gene transcription start sites are enriched in epigenetic marks, our selection of background variants ensures a good match when scoring variants is such regions. PINES also automatically annotates all variants with their distance to the nearest TSS. This annotation is not used by default in PINES, but the user can enable it via the source code.*

*The number of background variants corresponding to each chromosome correlates well with chromosome length (Supplemental Fig. S10). The background variants also display a good coverage of the autosomal genome (Supplemental Fig. S11), with annotations across sex chromosomes not yet included in the PINES model.*

*We have updated the section titled "Background variants and the null distribution" under Methods to include the above text.*

*The description of the actual PINES method is clearer, but again the authors could go into more detail. The chose the top 30 eigenvectors based on a scree plot of a PCA of the covariance matrix, but they could also state how much total variance is covered etc.*

*To account for potential differences in the annotation architecture of intronic and intergenic variants, we compute the background covariance matrix separately for these two variant classes. We noticed similar correlation structures, with the top 30 eigenvectors accounting for 53% of the variance for intronic variants, and 49% of the variance for intergenic variants.*

*We have included this text in the "Working with a high-dimensional correlated annotation space" section under Methods.*

The authors state that the all-1 annotation vector 1 is significant since it provides the direction of a point with maximal annotation load, and thus the greatest evidence for functionality. This assertion is not critical for understanding the method, but I think it is wrong -- it is mixing up the evidence of some biochemical mark such as a chromatin mark with biologically or medically relevant function (this was the same "controversy" surrounding claims that ENCODE showed that 80% of the genome has a function, which can be found by googling). The same comments applies further down to the claim "Smaller scores indicate greater evidence for functionality." I believe the authors should be more cautious with their interpretation of the data and avoid unsubstantiated statements about biological functionality -- I agree with the authors that this is plausible but there is no need to use language that is "hyped" to describe this nice method.

*We have updated our discussion around the all-1 annotation vector to address the reviewer's comment. We have also updated our wording throughout the manuscript to clarify that, while PINES aims to predict functionality based on functional genomic annotations, we are not making any prediction about the resulting biological effects. It is also important to note that all functional scoring methods that utilize epigenetic data follow the assumption that biochemical marks are informative to predicting functionality. Given the power of PINES to pinpoint known functional noncoding variants across a locus (Figure 2), we conclude that this assumption is reasonable.*

*The following is the updated paragraph from the Methods section:*

*"In PINES we are specifically interested in the angle between the projection of a variant of interest and the projection of the all-1 annotation vector 1. This vector is significant since it provides the direction of a point with maximal annotation load. Under the assumption that biochemical marks such as histone modifications are indicative of variant functionality, the presence of multiple epigenetic marks indicative of regulatory regions that are supported across multiple cell types provides the greatest level of functional evidence for the all-1 annotation vector."*

"Following the approach presented in [47], we additionally scale this angle by the length of the projected x vector,"

=> Again it would be helpful for the reader to summarize in one sentence the approach in 47 and to motivate why it is being used in PINES.

*Reference [47] presents an outlier detection method that addresses the curse of dimensionality by using an angle-based distance measure in addition to traditional Euclidean distance. We apply this principle in PINES, by scaling the angle between the projection of a variant of interest and the projection of the all-1 annotation vector by the length of the projected x vector.*

*We have added the text above to the "Working with a high-dimensional correlated annotation space" section under Methods to address this point.*

I would also like to see the source code and the data available to the reviewers -- the authors say it will be made available later.

*The source code and all annotations are now available through the PINES website (http://genetics.bwh.harvard.edu/pines/) under the GNU General Public License.*

The authors do not comment on the fact that an unknown proportion of the GWAS hits is likely to be functional with the rest being Tag SNPs. Since their method is posited upon being able to use functional data to prioritize variants, this needs to be discussed more. I am surprised that the authors did not attempt to test their method on some subset of GWAS hits that have been characterized as "functional" by some other method -- would this be a good idea? Can the authors show data on this?

*Examples for how PINES is able to correctly identify causal variants within "solved" loci are shown in Figure 2. For GWAS loci, we recommend that statistical fine-mapping be performed first, and then PINES can be used to prioritize among the variants in the resulting credible sets. Unfortunately, few GWAS credible sets have sufficient experimental follow-up to be used as gold standard (this would imply testing all variants in the credible set, not just the lead SNP).*

*Keeping this caveat in mind, our analysis in section "PINES detects signal at fine mapped GWAS risk loci" under Results applies PINES to a set of candidate causal variants that have been fine-mapped via PICS. We found that only 37% of the candidate causal noncoding variants identified by PICS also obtain a weighted PINES p-value <= 0.05. This highlights the ability of PINES to prioritize results from fine-mapping studies to further restrict the search space for causal variants and enable focused experimental follow-up.*

*We have included the above paragraph in the "PINES detects signal at fine mapped GWAS risk loci" section.*

**Second round of review**

**Reviewer 1:**

The authors showed great effort in the revision. The paper has been greatly improved. I just have a couple of remaining issues.

There is no references in the paper. All citations are marked [?]. This is not acceptable.

Not much detail is provided for Page 8, line 12, “Phenotype-based annotation weights were automatically assigned based on GWAS data”. I wonder whether any of these GWAS SNPs used to weight the annotations overlapped with any of the “fine mapped candidate causal noncoding variants”. The authors should clarify this because this is important to know.

In “Other methods” in the Methods section, the authors should include all methods they have compared with.

**Reviewer 2:**

The authors have made some revisions to the manuscript in response to some of the author points. I still have some concerns about the method's presentation and performance after revision.

* The references are not included in the manuscript. It appears the submitted version was not checked.

* The source code is distributed in a particularly inconvenient manner as it is bundled alongside the annotation files. This means it is a 10.5GB tar file that does not appear to be compressed. The source code should be separated from the annotations. The source code should be put on some sort of version control service such as GitHub so that authors can cite a certain version. The data files should be distributed via some mechanism that provides archiving and permanence such as figshare or zenodo.

* The negative evaluations are pretty weak. The authors note: "For example, throughout our manuscript we have used "unmatched" GWAVA scores in our comparisons (i.e. the background is selected from across the genome), since we noticed that using GWAVA scores based on more stringent background matching leads to reduced signal." This should not be surprising. Their evaluations reward the identification of cell-type relevant variants, even if they aren't functional. I am not sure that the evaluations in this paper are really stringent enough to warrant any sort of strong claims. I remain relatively skeptical as to the added value, as functional validation is lacking. The revision is somewhat helpful in that it specifies how weights can be fit, but still the evaluation is so limited in terms of comparison sets that I wouldn't know what method to pick afterwards. I am relatively confident that they can prioritize tissue-relevant variants. It seems this would be sufficient to return good scores by their evaluations. But I remain I'm much less confident that those variants will actually be functionally linked with a phenotype. For the most stringent evaluation of function, the MPRA, there appears to be essentially no difference between PINES, GenoCanyon, and GWAVA. This leads to additional concerns that their evaluations for PINES showing a substantial improvement across each other setting are flawed for the reasons already described.

* Initially I was concerned that this was a relatively simple approach presented as a complicated method. It still seems that way. For example, "We have also evaluated whether the low-rank approximation significantly improves the performance of PINES. Comparing PINES scores with and without the approximation on a set of 20,000 background variants suggests that this additional processing of the annotation covariance matrix has no significant impact on the results." If it doesn't matter, why include it at all? Without this, it's a relatively simple approach that appears to primarily count the independent sources of support for a variant when variants are not used for weighting.

**Reviewer 3:**

The authors have addressed my concerns and the revised manuscript is much improved.

**Authors’ response to reviewers:**

We thank the Reviewers for their comments, which we believe have further improved the manuscript. We respond to each of the Reviewers' comments below.

Reviewer #1: The authors showed great effort in the revision. The paper has been greatly improved. I just have a couple of remaining issues.

There is no references in the paper. All citations are marked [?]. This is not acceptable.

*We apologize for this omission: in this revision we will make sure that the manuscript is being compiled properly from our LaTeX source code.*

Not much detail is provided for Page 8, line 12, “Phenotype-based annotation weights were automatically assigned based on GWAS data”. I wonder whether any of these GWAS SNPs used to weight the annotations overlapped with any of the “fine mapped candidate causal noncoding variants”. The authors should clarify this because this is important to know.

*There is a partial overlap between the GWAS lead SNPs (used to construct the annotation weights) and the high-confidence (posterior probability > 0.25) PICS variants that were scored: about half of the PICS variants were also part of the GWAS SNPs. To evaluate the effect of this overlap on our comparisons it is important to note the following:*

*1.	GWAS lead SNPs are only used to extract a high-level representation of the relevant cell types. Supplemental Figs. S8-S11 depict all the information that is extracted from these variants and used in the subsequent weighted scoring step. These figures show that PINES upweights biologically relevant cell types for each disease (IBD: immune and GI, MS: immune and thymus, celiac disease: immune and GI, blood lipids: liver and adipocytes). Besides these cell type weights, no additional data is carried over to the scoring procedure.*

*2.	The GWAS lead SNPs are only used in an aggregated manner by evaluating the overlap of the entire set of variants with epigenetic annotations. Thus, no analysis is performed to learn features of individual GWAS lead SNPs.*

*To follow up on these observations, we tested whether the presence of variants used both as GWAS lead SNPs (to compute annotation weights) and scored PICS SNPs introduces any bias in our comparisons:*

*1.	Within the set of variants that achieved high PICS probabilities (posterior probability > 0.25), we evaluated whether also being a GWAS lead SNP resulted in more significant weighted PINES scores. We performed a Wilcoxon rank-sum test comparing the weighted PINES scores of PICS variants that are also GWAS lead SNPs versus those that are not GWAS lead SNPs, and observed no significant difference in the distribution of scores between these two categories (MS: p-value=0.7849, IBD: p-value = 0.7678, blood lipids: p-value = 0.6311, celiac disease: p-value =0.7302).*

*2.	We then re-ran all four disease-centered comparisons in Figure 3, but excluded all PICS variants that were also used in the computation of cell type weights. This way we only use variants that PINES has never seen in the cell type analysis step. The performance of PINES remains unchanged in comparison to the other methods: weighted PINES remains the best performing method based on the resulting AUROC values.*

*We have included the above analysis in the “PINES detects signal at fine-mapped GWAS risk loci” section under Results.*

In “Other methods” in the Methods section, the authors should include all methods they have compared with.

*We have added descriptions for all the methods included in our comparisons to the “Other methods” section.*

Reviewer #2: The authors have made some revisions to the manuscript in response to some of the author points. I still have some concerns about the method's presentation and performance after revision.

* The references are not included in the manuscript. It appears the submitted version was not checked.

*References are now added. Please see also our reply to Reviewer #1.*

* The source code is distributed in a particularly inconvenient manner as it is bundled alongside the annotation files. This means it is a 10.5GB tar file that does not appear to be compressed. The source code should be separated from the annotations. The source code should be put on some sort of version control service such as GitHub so that authors can cite a certain version. The data files should be distributed via some mechanism that provides archiving and permanence such as figshare or zenodo.

*To simplify the deployment of PINES we have uploaded the PINES source code to GitHub (https://github.com/PINES-scoring/PINES) and the annotation dataset to Zenodo (https://zenodo.org/record/1228512#.WuBft1MvyLI). The DOI for the annotation dataset is “10.5281/zenodo.1228512”. We include links to these resources on the PINES web portal, where users can also compute scores directly without extra computational overhead. We are currently working on computational improvements to the PINES software that will become available on GitHub as well as through the online portal.*

* The negative evaluations are pretty weak. The authors note: "For example, throughout our manuscript we have used "unmatched" GWAVA scores in our comparisons (i.e. the background is selected from across the genome), since we noticed that using GWAVA scores based on more stringent background matching leads to reduced signal." This should not be surprising.

*Regarding our use of “unmatched” GWAVA scores, throughout the manuscript our aim was to present results from the most stringent comparisons.*

Their evaluations reward the identification of cell-type relevant variants, even if they aren't functional. I am not sure that the evaluations in this paper are really stringent enough to warrant any sort of strong claims. I remain relatively skeptical as to the added value, as functional validation is lacking. The revision is somewhat helpful in that it specifies how weights can be fit, but still the evaluation is so limited in terms of comparison sets that I wouldn't know what method to pick afterwards. I am relatively confident that they can prioritize tissue-relevant variants. It seems this would be sufficient to return good scores by their evaluations. But I remain I'm much less confident that those variants will actually be functionally linked with a phenotype. For the most stringent evaluation of function, the MPRA, there appears to be essentially no difference between PINES, GenoCanyon, and GWAVA. This leads to additional concerns that their evaluations for PINES showing a substantial improvement across each other setting are flawed for the reasons already described.

*In the last revision we had carefully reworded potential overstatements regarding the power of PINES to predict biological effects. Instead, we clearly state that PINES predicts functionality based on the generally accepted assumption that biochemical marks inform on function. Given the power of PINES to pinpoint known functional noncoding variants across a locus (Figure 2), we conclude that this assumption is reasonable.*

*We disagree with the branding of MPRA as “the most stringent evaluation of function”, since this approach has multiple caveats:*

*1.	MPRA represents an artificial system, and the results can be affected by artifacts related to plasmid transfection and architecture of the construct [a recent publication (PMID: 29256496) nicely summarizes some of these caveats]. Additionally, MPRA relies on synthesized DNA sequences that are integrated in reporter gene plasmids and transfected into cell types of interest. Owing to the short length of synthesized fragments (150 bp in Tewhey et al.), MPRAs require accurate knowledge of putative regulatory region position and boundaries, which are not generally known.*

*2.	Based on the experimental setup, MPRA studies focus on just a few cell types. For example, the Tewhey et al. data (the basis for our MPRA panel in Figure 4) only reflects the regulatory activity in lymphoblastoid cell lines. In contrast, unweighted PINES scores (as well as most of the methods presented in Figure 4) reflect information across more than 100 cell types and tissues.*

*3.	Currently there is little replication data available for reported MPRA hits.*

*Due to the presence of these caveats, the Tewhey et al. manuscript itself mentions that “candidates identified through MPRA still require experimental validation” (for example through CRISPR/Cas9).*

*Based on these points we believe that well-characterized (through experiments and patient studies), highly-penetrant non-coding SNPs that cause human disease represent a good test set for methods such as PINES. We presented such examples for common noncoding variants in Figure 2, and showed that weighted PINES scores outperform all other methods (including unweighted PINES results).*

*To extend this analysis, we have additionally tested the performance of unweighted PINES to detect signal on a set of rare Mendelian variants curated by Smedely D. et al. (Am. J. Hum. Genet. 2016). Unweighted PINES was chosen for this analysis due to the strong diversity in phenotypes represented by this curated list. PINES achieved an AUROC of 0.87, indicating that even in an unweighted setting PINES can accurately identify validated functional noncoding variants.*

* Initially I was concerned that this was a relatively simple approach presented as a complicated method. It still seems that way. For example, "We have also evaluated whether the low-rank approximation significantly improves the performance of PINES. Comparing PINES scores with and without the approximation on a set of 20,000 background variants suggests that this additional processing of the annotation covariance matrix has no significant impact on the results." If it doesn't matter, why include it at all? Without this, it's a relatively simple approach that appears to primarily count the independent sources of support for a variant when variants are not used for weighting.

*While we appreciate the reviewer’s suggestion for a more concise presentation of the PINES methodology, we consider that the intuitive description of PINES as “a relatively simple approach that appears to primarily count the independent sources of support for a variant” is a strong over-simplification that fails to capture important features of our method. For example:*

*1.	The use of an angle-based distance measure as part of the scoring procedure, combined with a Cholesky transformation of the correlated annotation space, is unique among other methods, and contributes to the statistical power of PINES by addressing both correlations and the curse of dimensionality. A fine point here is that relying solely on a Cholesky transformation would negatively affect the performance of PINES by causing a reduction in significance levels for variants close to maximal annotation overlap.*

*2.	In addition, we employ the scaling presented in reference [49] to be sensitive to the angle-based distance measure as well as to a traditional Euclidean distance in the de-correlated annotation space.*

*These methodological design choices enable PINES to outperform other scoring methods even in the unweighted setting. We have expanded the “Working with a high-dimensional correlated annotation space” section under Methods and added a new supplemental figure (Figure S16) to include these points and add more details to the design of our method.*

*Additionally, we urge caution when evaluating a method based on its perceived complexity. As an example, Eigen-PC is a “simple” method primarily based on eigendecomposition of the annotation covariance matrix, yet it performs well.*

*In addition to what standard functionality scores such as Eigen-PC provide, the PINES methodology enables important new results:*

*1.	A weighted scoring customized to the user’s phenotype of interest. Throughout this manuscript we have highlighted the advantages provided by cell type-informed analyses, and we have shown (Supplemental Figs. S8-S11) that annotation weights computed by PINES map well to known biology.*

*2.	An approach to identify cell type-specific regulatory variants via a comparison of weighted and unweighted PINES scores (section “PINES identifies variants with highly cell type-specific annotation profiles” under Results). We have shown that this analysis provides a valuable starting point for experimental follow-up by highlighting relevant cell types.*

*Based on these improvements that PINES brings to noncoding variant scoring, we hope PINES will become a valuable resource for the interpretation of the noncoding human genome.*

*Regarding the use of a low-rank approximation, we have pointed out that this approach does not significantly affect PINES scores based on the current annotation dataset. As new annotations become available and are included in PINES, we expect the low-rank approximation to play a bigger role in reducing noise in our annotation covariance matrix. For example, new epigenetic annotations resulting from the efforts of the International Human Epigenome Consortium are already available for inclusion into PINES, so we wish to ensure that our method remains robust in the future.*

Reviewer #3: The authors have addressed my concerns and the revised manuscript is much improved.

**Third round of review**

**Reviewer 2:**

The authors present a revised version of this manuscript. They now provide source code and data in a reasonable way. I would like to applaud them for this.

The authors describe an evaluation that uses Smedley et al (2016) and achieves an AUROC of 0.87. I couldn't find this in the manuscript by looking for reference 18, nor could I find a comparison with other methods.

The authors argue against my previous point that MPRA is the most stringent evaluation of function by arguing that MPRA alone is not sufficient because one really needs to do additional experimental validation afterwards. Sure, this is true, but it remains the most stringent evaluation of function within their manuscript because they did not do the experimental validation.

The authors also note that omitting any step from the algorithm negatively affects the performance of PINES. However, they also say in the response to reviewers:

"Regarding the use of a low-rank approximation, we have pointed out that this approach does not significantly affect PINES scores based on the current annotation dataset."

This contrasts with language in the dataset that says: "We also account for noise in the annotation covariance matrix via a low-rank approximation..."

It seems that the authors are still claiming that this type of approach matters now within the manuscript. I also am having trouble squaring the cover letter with the response to reviewers with the manuscript. I find this confusing and don't know where the correct information can be found.

**Authors’ response to reviews:**

We thank the Reviewer for these comments, which we believe have further improved the manuscript. We respond to each of the Reviewers' comments below.

Reviewer #2: The authors present a revised version of this manuscript. They now provide source code and data in a reasonable way. I would like to applaud them for this.

The authors describe an evaluation that uses Smedley et al (2016) and achieves an AUROC of 0.87. I couldn't find this in the manuscript by looking for reference 18, nor could I find a comparison with other methods.

*We have added an updated analysis of the Smedley et al. data as well as an in-depth description of our approach to the "PINES prioritizes causal noncoding variants with high confidence" section under Results. This new paragraph reads as follows:*

*“In addition to testing the ability of PINES to prioritize among variants at these experimentally validated loci, we have also evaluated unweighted PINES on a manually curated list of rare noncoding variants that are connected to Mendelian disease [18]. In particular, variants were included in this list if the disease association was plausible on the basis of evidence such as familial cosegregation, experimental validation, or computational predictions. We scored the 38 reported autosomal variants connected to enhancer function and the 108 autosomal variants connected to promoter function via unweighted PINES as well as GWAVA, Eigen, CADD, LINSIGHT, DANN, and FATHMM-MKL. To avoid biases resulting from an uneven distribution of variant counts across the loci we perform a locus-centric comparison of scoring methods. In particular, for each locus and scoring method we computed the average p-value across all corresponding variants. We then recorded, for each locus, which method provided the most significant average p-value. In case of ties we recorded all methods that achieved the most significant average p-value. Supplemental Table S4 presents the number of loci for which each method achieved the most significant average p-value. We thus find that unweighted PINES outperforms all other methods on 15 loci, while the second-best methods only outperform on 10 loci.”*

The authors argue against my previous point that MPRA is the most stringent evaluation of function by arguing that MPRA alone is not sufficient because one really needs to do additional experimental validation afterwards. Sure, this is true, but it remains the most stringent evaluation of function within their manuscript because they did not do the experimental validation.

*We agree that in the absence of other experimental support MPRA may be a good tool to approximate function, yet the most stringent benchmarking of PINES in our manuscript is certainly against those loci where robust biological experimentation has been performed to unravel causal alleles (Figure 2).*

The authors also note that omitting any step from the algorithm negatively affects the performance of PINES. However, they also say in the response to reviewers:

"Regarding the use of a low-rank approximation, we have pointed out that this approach does not significantly affect PINES scores based on the current annotation dataset."

This contrasts with language in the dataset that says: "We also account for noise in the annotation covariance matrix via a low-rank approximation..."

It seems that the authors are still claiming that this type of approach matters now within the manuscript. I also am having trouble squaring the cover letter with the response to reviewers with the manuscript. I find this confusing and don't know where the correct information can be found.

*We have updated the Methods section of the manuscript to clarify our use of the low-rank approximation:*

*“In summary, this formula allows us to project annotation vectors of individual variants into a decorrelated annotation space, and compute their distance to the mean annotation vector in this space. We also account for differences between intronic and intergenic annotation profiles by considering these two types of variants separately, and enable the inclusion of weights into the scoring procedure. Finally, this formula accounts for potential noise in the annotation covariance matrix via a low-rank approximation, which will be particularly helpful in the future as more annotations are added to our dataset.”*
